# Analytical Performance of Nanobody-Based Immunoassay and Immunosensing Platforms for Bacteria and Toxin Detection: A Systematic Review

**DOI:** 10.3390/antib15010015

**Published:** 2026-02-21

**Authors:** Aya Jalil, Nadia Touil, Omar Nyabi, Elmostafa El Fahime, Sara Benlhachemi, Jean-Luc Gala, Khalid Ennibi, Karim Bakkouri, Abdelaziz Benjouad, Lamiae Belayachi

**Affiliations:** 1 Health Sciences Research Center, Higher School of Biomedical Engineering, College of Health Sciences, International University of Rabat, Technopolis Parc, Rocade of Rabat-Salé, Sala-Al Jadida 11100, Morocco; abdelaziz.benjouad@uir.ac.ma; 2 Center of Virology, Infectious and Tropical Diseases, Mohammed V Military Teaching Hospital, Faculty of Medicine and Pharmacy of Rabat, Biomedical Research and Epidemiology Unit (URBE), Rabat 10100, Morocco; ntouil2003@gmail.com (N.T.); kennibi@yahoo.fr (K.E.); 3 Mohammed VI Center of Research and Innovation, Mohammed VI University of Sciences and Health, Rabat 10100, Morocco; m.elfahime@gmail.com (E.E.F.); benlhachemi.sara@gmail.com (S.B.); kbakkouri@cm6.ma (K.B.); 4 Center for Applied Molecular Technologies (CTMA), Institute of Clinical and Experimental Research, Université Catholique de Louvain, 1200 Brussels, Belgium; omar.nyabi@uclouvain.be (O.N.); jean-luc.gala@uclouvain.be (J.-L.G.)

**Keywords:** bacterial toxin, bacteria, nanobody, single-domain antibodies, immunoassay, biosensor

## Abstract

**Background:** bacterial pathogens and their toxins present analytical challenges for rapid and specific detection, contributing to over 600 million cases of illness annually and worsening antimicrobial resistance (AMR). Conventional detection methods are useful but limited. Single-domain antibodies (sdAbs) offer alternative recognition elements with unique biochemical and engineering benefits, enabling the development of nanobody-based immunoassays and biosensing platforms that provide fast, highly selective, and reliable detection of bacterial pathogens and toxins in both food and clinical environments. **Objectives:** this systematic review assesses the analytical and functional performance of nanobody-based immunoassays and sensing formats for detecting bacteria and toxins across food and clinical samples. **Methods:** following PRISMA guidelines, major scientific databases were used to gather research, resulting in 32 eligible studies published between 2011 and 2025. **Results:** data collected included assay platforms, target bacteria and toxins, limit of detection, sensitivity, specificity, matrix recovery, and practicality. Risk of bias was evaluated using an adapted QUADAS-2 framework. The review shows that nanobody-based immunoassays have achieved high performance, thermostability, compatibility with genetic engineering, and versatile assay design. When combined with advanced transduction and signal amplification strategies, these systems contribute to the development of highly sensitive and user-friendly bioanalytical platforms for detecting bacteria and toxins. **Conclusions:** however, most studies relied on spiked samples and lacked large-scale validation, emphasizing the need for standardized benchmarking and real-world testing.

## 1. Introduction

Rapid and precise detection of bacterial pathogens and toxins remains a global priority for health and safety reasons, as they continue to threaten public health and economic stability, augmenting the risk of pandemic spread. The food industry, water and environmental quality control, and clinical diagnostics are key areas where swift detection of bacterial pathogens and protein toxins is essential [1,2]. Each year, unsafe food consumption causes approximately 600 million illnesses and 420,000 deaths worldwide, resulting in the loss of 33 million disability-adjusted life years (DALYs) [3]. In 2021, antimicrobial resistance (AMR) was responsible for an estimated 1.14 million deaths directly and contributed to 4.71 million worldwide; by 2050, this burden is expected to rise to nearly 1.91 million direct fatalities and 8.22 million overall [4]. The main bacterial agents responsible for most foodborne infections are *Escherichia coli*, *Salmonella enterica*, *Campylobacter jejuni*, *Staphylococcus aureus*, *Listeria monocytogenes*, and *Bacillus cereus* [4,5]. These pathogens can rapidly evolve, adapt, and grow under diverse conditions such as low or high temperatures, basic or acidic pH, a wide range of salinities, and various pressures [6]. Meanwhile, biological toxins are harmful substances produced by many organisms primarily for defense or survival. These toxic molecules can persist in different environments and cause adverse effects on other organisms, which may be exposed through injection, inhalation, ingestion, or skin contact [2,7].

The existing methods used to detect pathogens and toxins rely on conventional detection approaches that identify specific microbiological and biochemical markers, relying on three main strategies: plate culturing and colony counting techniques, polymerase chain reaction (PCR), and immunology-based assays [8]. However, they are often limited by lengthy processing times and the risk of mistimed sampling, which can lead to the misinterpretation of microbial behavior and growth dynamics [9]. Despite their limitations, these gold-standard techniques are still used successfully as effective detection tools, since they are often combined to obtain more reliable results [10]. Even so, there is a growing need for simple, rapid, sensitive, and cost-efficient technologies suitable for real-time, on-site monitoring. This has driven significant research interest in the development of immunosensing platforms for the detection of pathogenic microorganisms and toxins [11,12].

An immunoassay is a biochemical method for detecting and quantifying biomolecules in a sample using a specific antibody or aptamer that binds to its target antigen. The resulting signal, proportional to the antigen concentration, is generated through a label such as a radioisotope [13], chromophore [14], fluorophore [15], or enzyme [16]. Accordingly, immunoassays are classified as radio-, chromo-, fluoro-, or enzyme immunoassays, depending on the label used [17]. Enzyme-linked immunosorbent assay (ELISA) is the most heterogeneous enzyme immunoassay (EIA) technique used in clinical analyses and research [18]. There are four types of ELISA: direct ELISA (antigen-coated plate; screening antibody), indirect ELISA (antigen-coated plate; screening antigen/antibody), sandwich ELISA (antibody-coated plate; screening antigen), and competitive ELISA (screening antibody). The simplest protocol is the one used in the case of direct ELISA [19].

**Figure 1:**
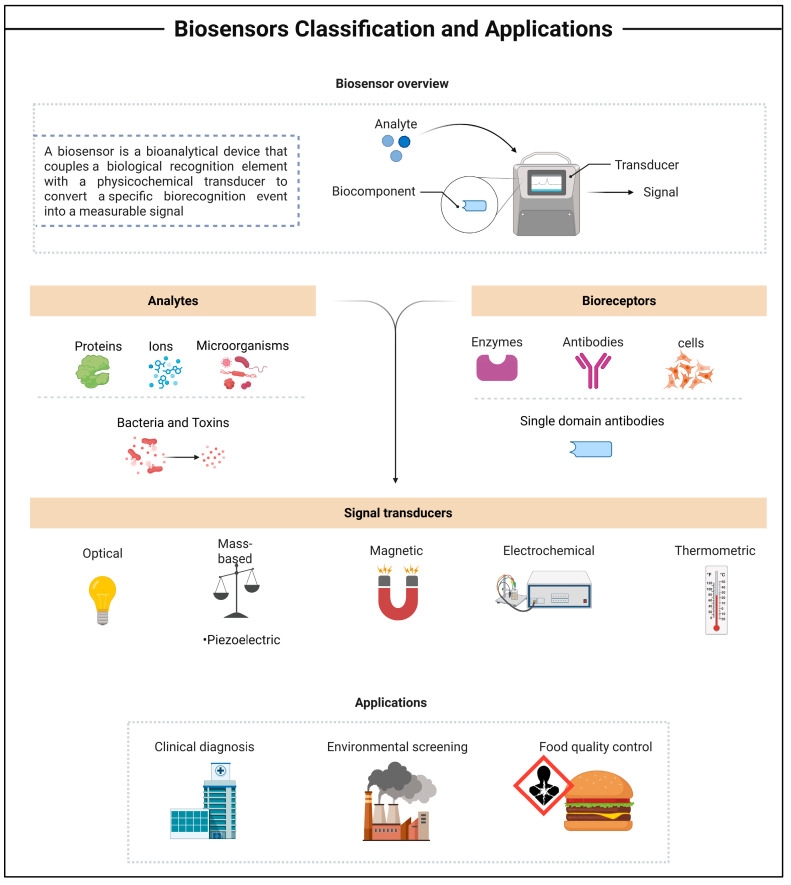
The basic principle of biosensor operation by biological recognition is converted into an electrical signal via a bioreceptor–transducer system. Created in BioRender. https://BioRender.com/xnsj9ek (accessed on 22 December 2025).

In recent years, research on biosensor technologies for detecting pathogenic microorganisms and toxins has rapidly grown. A biosensor is a bioanalytical device that couples a biological recognition element with a physicochemical transducer to convert a specific biorecognition event into a measurable signal (Figure 1). It consists of two core components: a bioreceptor that selectively binds the target analyte, and a transducer that translates this interaction into an electrical output [20]. Depending on the transduction mechanism, biosensors can be classified as electrochemical, optical, calorimetric, piezoelectric, acoustic, and electronic biosensors [21]. Electrochemical biosensors are the most extensively developed biosensing platforms, achieving significant commercial success, notably through amperometric glucose sensors widely used for diabetes monitoring. According to the International Union of Pure and Applied Chemistry (IUPAC) recommendations, an electrochemical biosensor is an integrated receptor–transducer device that generates selective quantitative or semi-quantitative analytical information through a biological recognition element [22].

Immunosensors are a major type of affinity biosensor category, and unlike traditional immunoassays, modern transducers enable label-free detection and quantification of the immune complex [17,23]. To avoid ambiguity, “immunoassay” refers to analytical tests that rely on antigen–antibody reactions, whereas “immunosensors” designates the complete biosensing device built on this immunorecognition principle [17]. In this latter, antigens or antibodies are immobilized to create the sensing element, and the resulting binding event is converted into a measurable signal by the transducer. The performance of these devices largely depends on the selectivity and the affinity of the antibody–analyte interaction. However, conventional antibodies present notable limitations, as their production requires complex preparation procedures, extended timelines, and usually involves animal sacrifice, raising both ethical and practical concerns [24,25]. Monoclonal antibodies (mAbs) are commonly produced using hybridoma technology, first reported in 1975 by Georges Köhler and Cesar Milstein [26]. This method involves the fusion of short-lived, antibody-producing B cells with immortal myeloma cells to generate hybridomas capable of continuous antibody secretion. Typically, spleen cells harvested from mice, rats, or rabbits that have been previously immunized with the antigen of interest are fused using polyethylene glycol (PEG). Following fusion, cells are cultured in a selective medium that allows only successfully fused hybridomas to survive. After several days, culture supernatants are screened for the presence of Ag-specific antibodies. Ab-producing hybridomas are then isolated and cloned, most often by limiting dilution, to obtain monoclonal populations secreting Ag-specific antibodies [27].

To address these drawbacks, novel approaches such as nanobody-based immunoassays and immunosensors have been developed. At the heart of these are “Nanobodies^®^” [28], a registered trademark of Ablynx N.V., designating single-domain antibody fragments derived solely from heavy chains and naturally present in camelids (Variable domain of Heavy chain of Heavy-chain-only antibodies, VHH), represent a promising alternative to conventional mAbs in the development of diagnostic tests. Thanks to their small size, high stability, and ease of production, they offer significant advantages in terms of sensitivity, specificity, and cost [24,29]. Unlike mAbs, VHHs are obtained by isolating VHH-encoding genetic sequences from lymphocytes collected after camelid (camels, llamas, alpacas) immunization with the desired antigen, which are cloned and recombinantly expressed in prokaryotic systems, enabling large-scale production.

The lack of the fragment (Fc) provides VHHs with a structural advantage, as it prevents nonspecific interactions with immunoglobulin-binding proteins on bacterial surfaces, thereby enhancing detection specificity. In addition, their remarkable stability under harsh conditions, including exposure to high temperatures or organic solvents, enables reliable on-site detection of water-insoluble hazards across diverse environments [30]. Moreover, their known amino acid sequences allow for straightforward genetic engineering, enabling fusion with other functional materials to create versatile constructs with multispecificity [31]. These features (Table 1) pave the way for the development of innovative and highly efficient nanobody-based immunoassays with broad applications in pathogen detection.

While narrative reviews have summarized individual nanobody applications, no systematic synthesis has critically assessed their analytical and practical performance across detection formats and target types. Therefore, this study systematically evaluates nanobody-based immunoassays for bacterial and toxin detection relevant to food and clinical biotechnology, comparing their sensitivity, specificity, limit of detection, and operational feasibility with conventional detection systems.

## 2. Materials and Methods

This systematic review was conducted in accordance with the Preferred Reporting Items for Systematic Reviews and Meta-Analyses (PRISMA) statement checklist 2020 [41]. The protocol was registered with the International Prospective of Systematic Reviews (PROSPERO registration number CRD420251088725).

### 2.1. Research Question

The proposed research question for this study was: “How do nanobody-based immunoassays perform across different detection platforms, in terms of sensitivity, specificity, and practicality for bacteria and toxin detection?”

### 2.2. PICO Elements

The research question was constructed based on the following PICO(S) schema, including:

P (Population): Samples potentially containing bacterial pathogens or toxins relevant to food safety, humans, or animals.

I (Intervention): Nanobody-based immunoassays as ELISA, lateral flow, electrochemical, colorimetric, biosensors, etc.

C (Comparator): Reference or conventional detection methods (culture, PCR, traditional/conventional antibody-based immunoassays).

O (Outcome): Diagnostic performance metrics such as sensitivity, specificity, limit of detection (LOD), accuracy, and practicality.

S (Study design): Diagnostic test accuracy (DTA) studies, experimental or applied studies that evaluate a new or modified assay by comparing its results to a reference standard, reporting metrics such as sensitivity, specificity, and limit of detection, and practical parameters such as time, cost, and ease of use.

### 2.3. Search Strategy

Database research was conducted using the PICO acronym (Population, Intervention, Comparisons, and Outcomes), which combines controlled vocabulary (MeSH terms) with free-text keywords. We searched the following databases for the relevant literature: PubMed, Scopus, PubMed Central (PMC), and ScienceDirect, from their earliest records up to the start of August 2025. The search was first conducted between July and 1 August 2025 inclusive, with search strategies tailored to the specific requirements of each database (Table S1 in Supplementary Materials). For ScienceDirect, instead of a structured search equation, relevant keywords were directly applied.

### 2.4. Inclusion and Exclusion Criteria

We employed the PICO framework for predetermined criteria for inclusion. The systematic review included studies that met all of the following criteria: (i) Reported original experimental or diagnostic performance data (in vitro, in vivo, or applied field studies) involving nanobody-based immunoassays. (ii) Eligible studies had to use VHHs either as capture reagents, detection elements, or both, and focus specifically on the detection of bacteria or bacterial toxins in clinical, and food samples. (iii) To ensure comparability, only articles that provided quantitative performance metrics, such as sensitivity, specificity, or limit of detection and were available in full text were considered. (iv) Publications were excluded if their targets were unrelated to bacteria or bacterial toxins, such as studies on viruses, fungi, mycotoxins, aflatoxins, cyanobacteria, parasites, or cancer. (v) Studies were also excluded if they did not involve nanobody-based assays, did not constitute diagnostic or immunoassay research, or failed to report performance outcomes. Furthermore, (vi) research articles in languages other than English or French, as well as (vii) inaccessible texts, were excluded.

### 2.5. Data Extraction

Articles were independently searched and screened by two authors based on the inclusion and exclusion criteria. After evaluation of titles and abstracts, duplicates were removed, and potential studies fulfilling the inclusion criteria were chosen for this review. The screening process and data extraction were performed using Microsoft Excel 365. The following data were extracted: (i) target pathogen and toxin, (ii) type of immunization, (iii) nanobody-based immunoassay platform used and its comparator, (iv) capture and detection of antibody/nanobody, (v) nanobody property, (vi) limit of detection, sensitivity, and specificity, (vii) recovery (matrix effect), (viii) sample matrices tested and spiked samples, and (ix) practicality of the platform used.

### 2.6. Risk of Bias Assessment

The QUADAS-2 tool was used to assess the methodological quality of all studies included in this systematic review [42]. Risk of bias was assessed independently by two reviewers using the QUADAS-2 tool, adapted to the context of nanobody-based immunoassays. The four domains evaluated were sample selection, the index test, the reference standard, and flow and timing. Each domain was rated as low, high, or some concerns, and judgments were recorded with supporting justifications.

## 3. Results

### 3.1. Studies Identified by Database Searches

**Figure 2:**
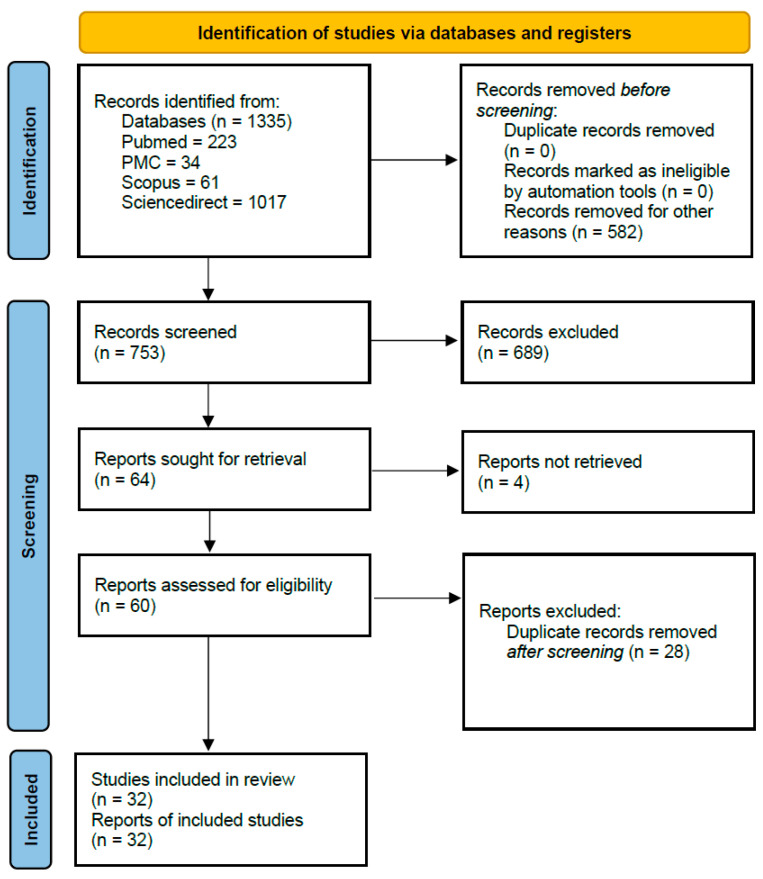
PRISMA 2020 flow diagram for new systematic reviews of database searching and study selection and identification.

The results of the search and selection process are presented in Figure 2. A total of 1335 records were identified through database searches, including 223 from PubMed, 34 from PMC, 61 from Scopus, and 1017 from ScienceDirect. After removing 582 records that were ineligible prior to screening, 753 records were screened. Of these, 689 were excluded based on title and abstract review. Sixty-four reports were sought for retrieval, of which four could not be obtained. The remaining 60 full-text articles were assessed for eligibility, leading to the exclusion of 28 duplicates identified at this stage. Ultimately, 32 studies met the inclusion criteria as shown (Figure 2) and were incorporated into the systematic review.

### 3.2. Characteristics of the Included Studies

**Figure 3:**
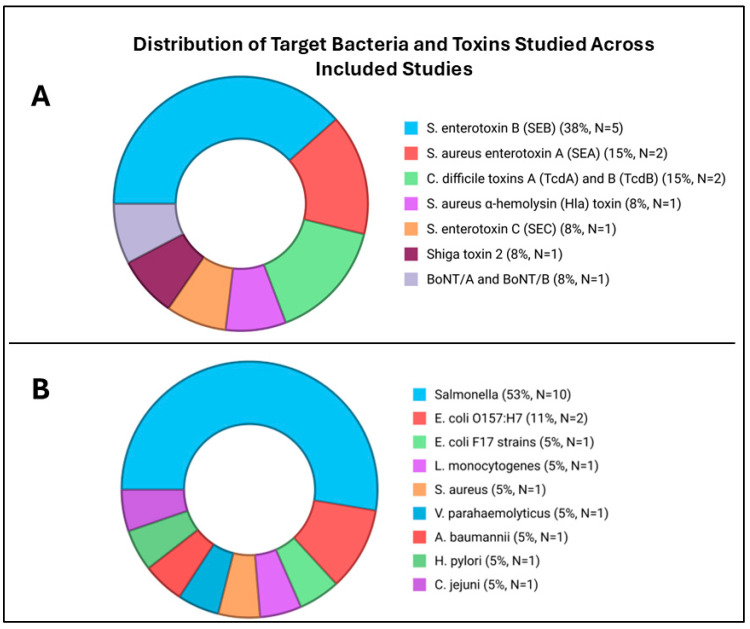
Distribution of target bacteria and toxins investigated in the dataset. (**A**) Toxins analyzed in the included studies. (**B**) Bacterial species examined across the dataset. Each pie segment represents the number of studies focusing on a specific target. Recurrent targets include *Salmonella* (*S. enterica serovar Enteritidis* (*S. enteritidis*), *S. enteritidis* and *serotypes*, *S. typhimurium*, *S. london*, *S. paratyphi* B, *S. hadar*), *Staphylococcus aureus enterotoxins* (SEA, SEB, SEC), and clinically relevant pathogens such as *E. coli O157:H7*, *Clostridium difficile* toxins, and *Listeria monocytogenes*. The distribution highlights research emphasis on foodborne pathogens and bacterial toxins with major public health impact. Created in https://BioRender.com (accessed on 29 December 2025).

The selected studies were original experimental works that focused on the development of nanobody-based assays for detecting pathogens present in various samples, including food matrices, stool specimens, clinical isolates from hospital patients, controlled laboratory samples, and biological fluids. The publication years of the selected studies ranged from 2011 to 2025, reflecting progressive advances in assay platforms. Of the 32 included studies, 19 (59.37%) investigated nanobody-based immunoassays for bacterial detection (Figure 3B), whereas 13 (40.62%) focused on toxin detection (Figure 3A).

**Figure 4:**
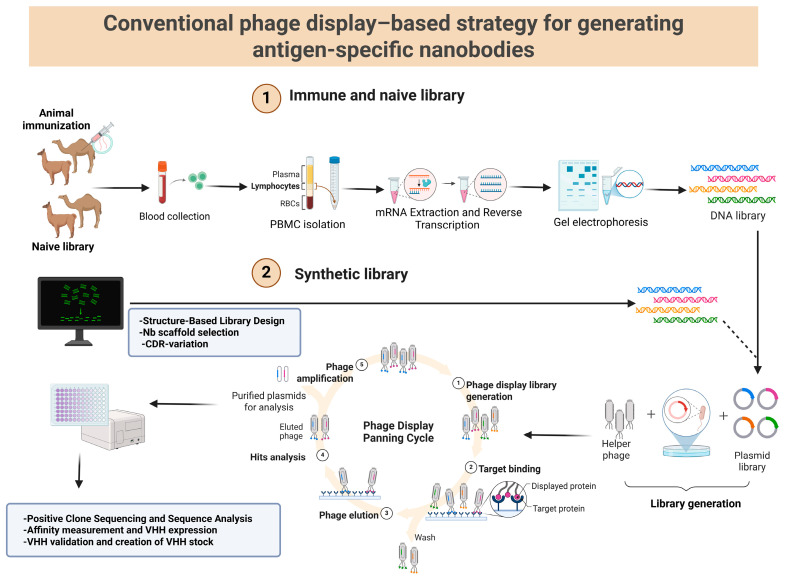
Schematic overview of the nanobody generation and selection process for the development of nanobody-based immunoassays and immunosensors. Created in BioRender. https://BioRender.com/0wyy0hl (accessed on 23 December 2025).

Most VHHs were derived from immune camelid libraries; however, some studies used naïve or synthetic libraries (Figure 4), indicating a gradual transition toward animal-free production. Several studies tested multiple matrices, for example, milk and pork, juice and honey, etc. Dairy products were the most tested matrices (59.4%), followed by meat and animal-derived products (43.8%). Clinical or human/animal biological samples were included in 25.0% of studies, while 18.8% evaluated plant-based products. Lastly, few studies (6.3%) relied solely on artificial laboratory systems.

Across the studies included, the choice of analytical platforms favored established assay formats, with fewer studies investigating emerging or alternative biosensing approaches. ELISA-based assays dominated the literature, whereas lateral flow immunochromatographic formats were only modestly explored. Electrochemical biosensors and nanozyme- or photothermal/fluorescence-based approaches remained underrepresented, despite their potential advantages for sensitivity and rapid detection. Multiplex strategies were notably scarce, with only a single study employing a Luminex-based assay.

While early assays relied on polyclonal or monoclonal capture antibodies paired with nanobody probes, several recent ones employed fully nanobody-based designs or engineered constructs such as nanobody–HRP fusions, phage-displayed VHHs, or nanobody–aptamer hybrids, demonstrating the versatility of VHH domains. As for the analytical performance, it varied considerably as shown in Table 2 and Table 3. Platforms such as phage-mediated chemiluminescent ELISA (P-CLISA) and dual-mode colorimetric/photothermal assays enhanced detection by up to 10- to 100-fold compared with conventional ELISAs. Across the included studies, analytical sensitivity was consistently reported using qualitative measures (LOD, regression values); however, only two studies provided a quantitative percentage value [43,44]. Specificity was high across studies, with minimal cross-reactivity reported qualitatively, although some exceptions occurred due to antigenic similarity (*S. typhimurium* vs. *S. paratyphi*). Analytical parameters such as recovery, LOD, and relative (RSD) were reported with numerical values across the reviewed studies, as shown in the Supplementary Materials Tables S2 and S3, ensuring a reliable comparison of assay performance.

### 3.3. Methodological Quality of Included Studies

To assess the methodological quality and risk of bias of the included studies, the QUADAS-2 tool was employed. Since our review focused on diagnostic assays applied to food matrices, stool specimens, clinical isolates, controlled laboratory samples, and biological fluids, rather than patients, the first domain, “patient selection,” was adapted to “sample selection” (Table 4). This adaptation evaluated whether studies used naturally contaminated collected samples or relied only on artificially spiked matrices, which may overestimate diagnostic performance. The second domain, index test, examined the nanobody-based assay under evaluation (ELISA, LFIA, electrochemical, biosensor, etc.), assessing whether assay conditions, cut-offs, and thresholds were pre-specified and if blinding to reference results was performed. The third domain, reference standard, considered the appropriateness of the comparator methods (culture, PCR, or commercial antibody-based immunoassays). The fourth domain, flow and timing, assessed whether all samples received both index and reference tests, whether the interval between tests could introduce bias, and if any samples were excluded from the analysis. Applicability concerns were also adapted, focusing on whether the study design and sample type aligned with the review question. This tailored application of QUADAS-2 ensured that the tool was relevant to nanobody-based immunoassays, allowing for the systematic identification of methodological strengths, weaknesses, and risks of bias across the 32 included studies.

**Figure 5:**
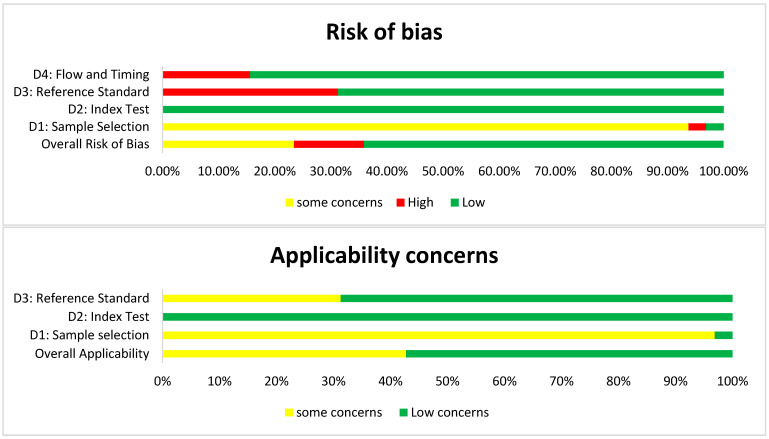
Risk of bias and applicability concerns summary: QUADAS-2 bias assessment and QUADAS-2 applicability assessment items presented as risk of bias and applicability concern graph percentages across all included studies.

**Figure 6:**
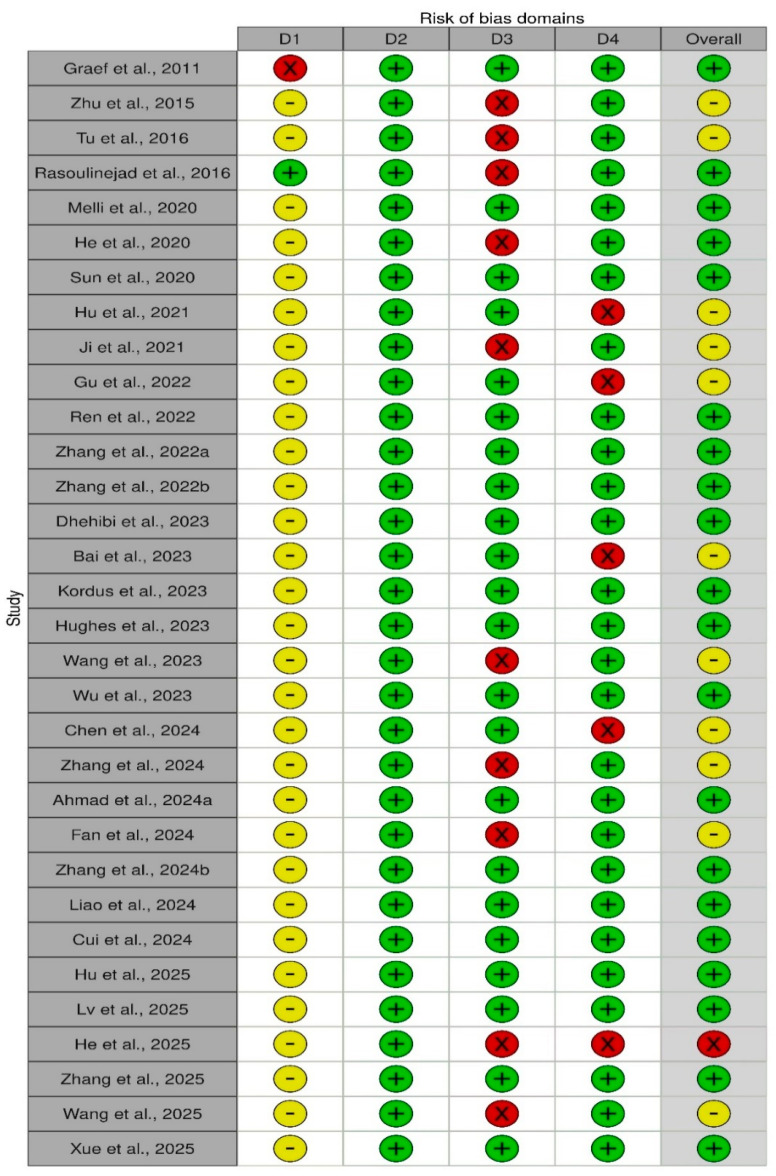
Methodological quality summary table of risk of bias summary: review authors’ judgements about each domain for each included study [75]. With red spotlight: high, yellow: some concern, and green: low. Domains: D1 for sample selection, D2 for index test, D3 for reference standard, and D4 for flow and timing [43,44,45,46,47,48,49,50,51,52,53,54,55,56,57,58,59,60,61,62,63,64,65,66,67,68,69,70,71,72,73,74].

The results of the quality assessment were summarized in (Figure 5 and Figure 6). The assessment was carried out using the criteria presented in (Table 4). For sample selection, risk of bias, and applicability, the results were moderate, as nearly all studies relied on spiked food and stool matrices rather than naturally contaminated samples. Although convenient for controlled proof-of-concept testing, they are not representative of real-world contamination scenarios, raising questions about the generalizability of reported sensitivities and limits of detection. For the index test, the risk of bias was low in all cases, as assay protocols were well-described, cutoffs were pre-specified, and performance metrics were systematically reported. Applicability concerns for this domain were minimal, as nanobody immunoassays directly addressed the review question. Regarding the reference standard, most studies used culture, PCR, or commercial ELISAs, which represent well-established comparators, leading to predominantly low risk of bias and low applicability concern. However, a subset of studies (31.25%) did not include a clear comparator, which increases both the risk of bias and concerns over applicability. Finally, for flow and timing, most studies processed samples immediately after preparation and analyzed all included replicates, resulting in an overall low risk of bias. Nevertheless, some reports omitted details on excluded replicates or did not clarify the timing between the index test and reference standard, contributing to high risk in a minority of cases. Overall, these findings suggest that while nanobody-based immunoassays are technically robust and aligned with the review’s diagnostic question, their broader applicability to real-world food safety and clinical diagnostics remains limited by study designs that predominantly relied on spiked rather than naturally contaminated samples.

## 4. Discussion

Antibodies are regarded as some of the most effective biomolecules for detection [76,77,78]. Currently, there is an increasing demand to enhance traditional antibodies, and VHHs have emerged as promising alternatives in the field of diagnosis. Due to their relatively low molecular weight and simple structure, VHHs are easier to adapt and incorporate into various detection systems or therapeutic strategies. Based on these advantages, this study demonstrates how the rational design and epitope-guided pairing of VHHs can be systematically translated into multiple diagnostic formats, enabling sensitive, rapid, and cost-effective detection while highlighting the versatility of VHHs across different assay architectures.

### 4.1. Technical Features of Nanobody-Based Platforms

Across the reviewed literature, the principal advantage of VHHs is their recombinant nature, which allows for efficient genetic engineering and flexible assay design. By contrast, reported physicochemical parameters such as thermal stability and expression yield are variable and should not be regarded as generally representative. Crucially, analytical quality relies on nanobody monodispersity, yet reagent-level quality control was rarely reported in the analyzed studies, limiting confidence in the robustness and reproducibility of the resulting immunoassays. Despite these limitations, certain context-specific functional advantages of VHHs were clearly demonstrated in individual studies. The lack of Fc regions reduced nonspecific binding, enhancing specificity in complex matrices prone to interference. Ji et al. (2021) demonstrated high specificity for *SEC*, with negligible cross-reactivity toward structurally related *enterotoxins* (*SEA*, *SEB*), *S. aureus Protein A* (*SpA*), or whole *S. aureus* strains, as evidence by absent signals in blank and non-target controls, demonstrating that the VHH retained precise antigen recognition, ensuring reliable and selective detection of *SEC* in complex samples [70]. Similarly, Ren et al. (2022) applied a broad spectrum of *Salmonella* nanobody (Nb-01) in a streptavidin-bridged sandwich ELISA (SAB-ELISA), enabling simultaneous detection of five major *Salmonella* serovars (*S. enteritidis*, *S. typhimurium*, *S. london*, *S. paratyphi* B, and *S. hadar*) with strict specificity and precision and with no cross-reactivity [50]. These findings affirm VHHs’ superior selectivity, supporting their role in reliable, multiplexed pathogen detection while underscoring the need for reagent-level validation to generalize beyond assay-specific designs.

Functional engineering broadens nanobody versatility across diverse platforms, from enzyme fusions to nanoparticle conjugates. Lv et al. (2025) designed an HRP-fused VHH targeting *BoNT/A* and *BoNT/B* for rapid ELISA detection by adding a Trx tag at the N-terminus, a 6 × His tag at the C-terminus, and fusing them with HRP [44]. While Wang et al. (2023) enhanced detection sensitivity via AuNP conjugation in a one-step label-free colorimetric strategy using M13 bacteriophage-displayed nanobody (phage-Nb) for *V. parahaemolyticus*; the thiolated phage-VHH induced AuNP aggregation, prevented by target binding to yield a visible color shift, achieving rapid detection (<100 min) with a visual detection limit of 10^4^ CFU/mL and a quantitative limit of 10^3^ CFU/mL without cross-reactivity [55]. Building on this, Wang et al. (2025) employed streptavidin–biotin “Molecular Velcro” for oriented conjugation of a biotinylated VHH to streptavidin-coated AuNPs, yielding Au/SA@Bio-Nb probes with higher stability and affinity [49]. Coupled with high *E. coli* expression yields (2.7–10 mg/L), these strategies underscore low-cost scalability [68]. Overall, these findings confirm that VHHs are not only biophysically resilient but also highly adaptable, engineerable biomolecules that can be utilized on a wide variety of diagnostic platforms. Their combination of stability, solubility, and specificity underpins their growing role as recognition elements in food safety and clinical biosensing.

### 4.2. Analytical Performance and Field Applicability

Nanobody-based assays often enable shorter time to detection, primarily due to improved antigen capture kinetics, higher functional surface density, and enhanced robustness compared to conventional antibody formats. Several assays achieved detection within 15 min and enhanced robustness versus conventional antibody formats. The gold/streptavidin–biotin nanobody-based lateral flow immunoassay (Au/SA@Bio-Nb-LFIA) detected *S. typhimurium* in milk, juice, and pork with LODs of *S. typhimurium* in milk: 10^3^ CFU·mL^−1^, juice: 10^3^ CFU·mL^−1^, and pork: 10^4^ CFU·mL^−1^, recoveries of 81.23–105.01% (RSD = 1.96% to 6.20%), and matrix tolerance [49]. Similarly, the photothermal KNb-DITS biosensor identified *S. typhimurium* within 20 min. They combined flower-like 3D KMO@Au composites with mini-structure–high-stability–high-affinity Nb9 to form KMO@Au@Nb9 photothermal immune probes, which can be observed by the naked eye and colorimetrically analyzed by the ImageJ software. To determine the specificity of the assay, the KNb-DITS was examined with nine other foodborne pathogens, including *S. paratyphi*, *S. london*, *S. enteritidis*, *S. hadar*, *S. aureus*, *C. coli*, *C. albicans*, *E. coli*, and *L. monocytogenes*. No significant signal changes were observed upon addition of other bacteria with 10^8^ CFU mL^−1^ except for *S. paratyphi*, which shares lipopolysaccharides (LPSs) with *S. typhimurium*. The high selectivity of the biosensor is attributed to the high specificity of the nanobody, which avoids the nonspecific binding of the Fc fragment. Another study of Zhang et al. (2022) [51] developed a P-CLISA for the detection of *S. typhimurium*, using soluble Nb9 as the capture nanobody and phage-displayed Nb1 as the detection element. Four *S. typhimurium*-specific VHHs were expressed and characterized, showing high affinity and pronounced thermostability, after which Nb1 and Nb9 were selected through epitope mapping. Compared with a conventional double-nanobody ELISA, phage-mediated signal amplification resulted in an approximately 100-fold improvement in sensitivity. The integration of a chemiluminescent readout further enhanced analytical performance, yielding an LOD of 3.63 × 10^3^ CFU/mL over a linear range of 5.1 × 10^3^–1.2 × 10^6^ CFU/mL. The assay exhibited negligible cross-reactivity with non-target foodborne pathogens and enabled the detection of fewer than 10 CFU/mL in food samples following short pre-enrichment. Overall, this study shows that assay performance is influenced not only by the intrinsic properties of the binding molecules but also by the overall assay architecture. High-affinity and thermostable VHHs alone were insufficient to achieve high analytical sensitivity; instead, significant improvements were obtained through optimization of the detection strategy, particularly via phage-mediated amplification and chemiluminescent readout, underscoring the central role of assay design in determining sensitivity [51].

For toxin detection, Lv et al. (2025) [44] established two ELISA methods and a time-resolved fluorescence immunochromatography assay (TRFICA) for *BoNT/A* and *BoNT/B* by using clinical symptoms as the “gold standard.” A total of 49 blood samples were tested on patients diagnosed with botulinum poisoning, and 48 were positive for *BoNT*, with a sensitivity of up to 98%. Moreover, all 48 positive patients were poisoned by type A *BoNT*. Among the 30 negative serum samples, TRFCIA exhibited negative results in 29 samples. Only one sample showed a false positive, with a specificity of 96.7%. These results showed that the TRFCIA method can successfully be used to detect *BoNT* poisoning in clinical samples. Compared with ELISA 1, ELISA 2 has a lower detection cost, and the detection time was greatly shortened from 100 min for ELISA 1 to only 70 min for ELISA 2, and for the TRFICA method, it was 15 min. However, the LOD for ELISA was as low as 0.17 ng/mL, and that for TRFICA was 0.05 ng/mL. In this paper, marked differences in sensitivity, speed, and robustness were observed depending on the assay format and signal transduction strategy, with ELISA and TRFICA exhibiting distinct performance profiles despite relying on the same nanobody binders. The incorporation of tandem nanobody designs, reporter fusions, and lanthanide-based fluorescence significantly enhanced detection sensitivity and reduced assay time, demonstrating that assay architecture and signal amplification strategies play a decisive role in determining analytical outcomes [44].

Many other papers reported results like standard ELISA-based formats that required 3 to 4 h, including incubation and wash cycles. Ji et al. (2021) [70] created an ELISA based on VHHs (sandwich VHH-ELISA) to detect *SEC* in dairy products without the influence of *SpA*. Eleven VHHs against *SEC* were identified in three biopanning steps. Their activity was assessed using indirect ELISA; their LOD ranged from 0.13 to 11.63 ng/mL. The specificity was tested by analyzing alongside *staphylococcal enterotoxins* A, B, and C. ELISA exhibited no cross-reactivity with *SEA*; meanwhile, a cross-reaction was shown with *SEB* and *SEC1*, indicating partial antigenic overlap due to a high sequence homology, where SEC shares at least 65% amino acid with *SEB* and over 95% with *SEC* variants. Hence, this method exhibits a broad quantitative range between 4 ng/mL and 250 ng/mL with an LOD = 2.47 ng/mL [70]. Wu et al. (2023) [67] introduced a nanobody-armed photothermal lateral flow immunoassay (NLFIA), in which VHHs act as the “umbrella of tolerance”, improving the stability of LFIA, complemented by Au core–petal nanoparticles (CPNs), a photothermal material synthesized by a polydopamine (PDA)-assisted two-step method, to enhance the sensitivity of the readable signal detecting *SEB*. NLFIA offered better cLOD of 1.68 ng/mL for colorimetric mode and 0.58 ng/mL for photothermal mode, higher environmental tolerance, and greater specificity, almost completely avoiding the interference of SpA in the detection of SEB [67].

Cost-effectiveness and scalability are major advantages. First of all, the use of *E. coli* represents one of the most widely used systems of expression of VHHs other than *Pichia pastoris*, plant systems such as tobacco plants and *Arabidopsis thaliana*, and mammalian cells [62]. Owing to its well-known genetic properties, *E. coli* remains the preferred expression host due to its low production cost, ashort fermentation cycle, easy cultivation, convenient operation, and high recombinant yield [79]. Secondly, platform design further influences affordability. Zhang et al. (2024) [63] developed a double-nanobody “RANbody” sandwich ELISA, which eliminates the need for secondary antibodies by possessing both recognition and catalytic capabilities, thereby reducing both cost and time compared to traditional sandwich ELISA. The platform maintained a low LOD of 10 ng/mL for α-hemolysin with excellent specificity [63].

Multiplexed detection represents another innovation. Ren et al. (2022) designed a SAB-ELISA based on self-paired VHHs for monitoring multiplex Salmonella serogroups within 180 min and LODs: 6.31 × 10^3^ CFU/mL of *S. typhimurium*, 9.15 × 10^3^ CFU/mL of *S. enteritidis*, 4.23 × 10^3^ CFU/mL of *S. london*, 7.31 × 10^3^ CFU/mL of *S. paratyphi*, and 7.25 × 10^3^ CFU/mL of *S. hadar* [50]. Another group created a dual-signal amplified LFIA (D-LFIA) platform integrating nanobody-engineered magnetic quantum dot nanocomposites Fe_3_O_4_@SiO_2_@TQD for the rapid and quantitative detection of *S. enteritidis*, *L. monocytogenes*, and *C. jejuni*. VHHs served as additional probes in the dual-probe mode to enhance the sensitivity of the Fe_3_O_4_@SiO_2_@TQD-based LFI. The D-LFIA system showed excellent analytical performance, with an LOD of 38, 125, and 47 CFU/mL for *S. enteritidis*, *L. monocytogenes*, and *C. jejuni*, respectively, representing a 32- to 54.9-fold improvement over conventional single-probe LFIA, with rapid detection within 13 min [57].

The common thread across these studies is that VHHs are used as enabling elements to overcome key limitations of conventional immunoassays and function as flexible and scalable recognition modules whose full analytical potential is realized when coupled with optimized detection formats, signal transduction chemistries, and platform-level innovation. Across ELISA, LFIA, photothermal, chemiluminescent, and multiplexed formats, VHHs provide high specificity, robustness against matrix interference, and compatibility with recombinant expression in low-cost systems. However, improvements in sensitivity, assay time, multiplexing capacity, and affordability are largely driven by innovative assay architectures, including signal amplification strategies, reporter-fused VHHs, photothermal or fluorescent nanomaterials, and dual- or self-pairing designs.

### 4.3. Comparative Perspective with Conventional Antibody-Based Immunoassays

The comparisons between nanobody-based immunoassays and conventional antibody-based formats were conducted using a combination of conceptual, indirect, and parallel benchmarking approaches, rather than head-to-head evaluations. Several studies explicitly addressed the advantages of VHHs, particularly the lack of Fc-mediated interactions, which reduced nonspecific binding in complex matrices [67,70]. In other papers, the comparative perspective was primarily indirect. Ren et al. (2022) benchmarked their streptavidin-bridged nanobody ELISA for multiplex *Salmonella* detection against previously reported mAb/pAb ELISAs, emphasizing broader serovar coverage and reduced assay complexity [50]. Likewise, Wang et al. (2025) compared their oriented Nb–gold nanoparticle probes with conventional antibody adsorption strategies described in earlier LFIA studies, reporting enhanced stability and affinity, though without an IgG control implemented using the sale probe configuration [49]. Nb-based platforms were also compared against commercial kits or gold-standard diagnostic methods. For instance, the VHH-based *shiga toxin* capture ELISA was evaluated alongside commercial toxin detection kits and PCR/culture methods, showing comparable or improved sensitivity in clinical samples, thereby supporting diagnostic relevance but not isolating binder type as the sole variable [72]. Similarly, Lv et al. (2025) validated Nb-based ELISA and TRIFCA formats for *botulinum neurotoxin* detection using clinical diagnosis as the reference standard, demonstrating faster detection and reduced cost relative to traditional ELISA workflows, with improvements driven in part by reporter fusion and time-resolved fluorescence rather than binder substitution alone [44]. Other studies emphasized platform-level enhancements that complicate direct comparison with conventional antibodies. Phage-displayed Nb assays and dual-signal or nanoparticle-assisted LFIAs frequently reported sensitivity improvements of one to two orders of magnitude compared with conventional Ab-based formats described in the literature; however, these gains were closely linked to multivalency, signal amplification, or enrichment strategies rather than to demonstrated differences in intrinsic binding affinity between VHHs and IgGs [55,56,57]. Conceptual comparisons were also common in studies employing RANbody or Nb–enzyme fusion formats, which highlighted reduced assay time, elimination of secondary antibodies, and simplified workflows compared with traditional sandwich ELISAs, while stopping short of direct matched comparisons with IgG-based assays under identical conditions [63]. However, because most comparisons are indirect, or benchmarked against commercial kits rather than constructed IgG controls under the same conditions, definitive attribution of enhanced performance to intrinsic nanobody properties remains limited. This highlights the need for future studies to incorporate rigorous head-to-head benchmarking against conventional antibodies using a matched assay architecture.

### 4.4. Key Findings

Nanobody-based immunoassays exhibit consistently strong analytical performance in the detection of bacteria and toxins, particularly in foodborne infection contexts. Sandwich ELISA was the most employed platform, followed by LFIA, electrochemical sensors, and dual-mode assays (colorimetric/fluorescence or photothermal/ELISA), which enhanced detection sensitivity. Limits of detection varied from picogram levels for toxins to 10^3^–10^5^ CFU/mL for bacteria, often requiring enrichment for lower concentrations. Specificity was uniformly high, largely due to the absence of Fc-mediated cross-reactivity commonly seen in conventional antibodies. Recovery rates in spiked matrices yielded between 70–110%, with a coefficient of variation below 15%, confirming assay precision. VHHs were mostly derived from immunized camelids (llamas, alpacas), though naïve and synthetic libraries also proved effective. Overall, these results position VHHs as robust molecular recognition elements for diagnostic and biosensing technologies.

### 4.5. Limitations

Despite promising performance, several limitations were identified. Nearly all studies used spiked samples rather than naturally contaminated samples, which limits their real-world applicability and validity. Some studies did not include comparator reference methods such as culture, PCR, or commercial ELISA, raising concerns about diagnostic benchmarking. Additionally, in many studies, nanobody-based platforms were not directly benchmarked against IgG-based counterparts under identical conditions, limiting definitive conclusions regarding their comparative analytical advantage.

Beyond essay-level validation, limited attention was paid to the quality of the nanobody-based reagents. In most studies, nanobody functionality was inferred from successful signal generation within the final immunoassay format, and independent confirmation that the produced nanobody-based reagents was rarely reported. Reagent characterization was typically restricted to expression yield or SDS-PAGE analysis, which does not exclude aggregation, partial misfolding, or heterogeneity, particularly for engineered constructs such as enzyme fusions, multivalent VHHs, phage displayed formats, or nanobody–nanomaterial conjugates. This lack of reagent-level validation complicates the attribution of assay performance to intrinsic nanobody properties rather than to platform-level amplification or multivalency effects.

Sensitivity reporting was also limited; only two studies provided explicit sensitivity values, while others inferred assay performance from LOD, linear range, or recovery data. Furthermore, validation was mainly confined to controlled laboratory conditions with small sample sizes; few studies assessed long-term stability, batch-to-batch reproducibility, or field-level robustness. These limitations highlight the need for more comprehensive and standardized analytical validation frameworks.

### 4.6. Research Gaps

Future research should address several clear gaps. There is an urgent need for validation using naturally contaminated samples across various matrices, including food and clinical diagnostics, to confirm applicability beyond laboratory settings. Standardized reporting of analytical performance metrics (sensitivity, specificity) should be systematically reported to enable comparability and meta-analysis. Comparative studies of gold-standard diagnostic methods and commercial kits are lacking and essential for regulatory and industry uptake. In addition, multiplexed detection assays remain underdeveloped, despite their high relevance for simultaneous bacterial pathogen and toxin screening in food safety and clinical contexts. Finally, cost-effectiveness, large-scale production, and integration into point-of-care or field-deployable devices represent underexplored frontiers that will determine real-world adoption. Addressing these gaps will be key to translating nanobody-based immunoassays into reliable, standardized, and globally accessible bioanalytical technologies.

## 5. Conclusions

To the best of our knowledge, this is the first systematic review focused on nanobody-based immunoassays for the detection of bacterial pathogens and protein toxins. Analysis of 32 studies highlights the engineering flexibility of VHHs as recombinant recognition elements, enabling their integration into diverse biosensing platforms. Reported gains in specificity and operational robustness are largely associated with assay architecture and platform design, rather than intrinsic physicochemical superiority of VHHs. Importantly, reagent-level quality control, particularly monodispersity assessment, was rarely reported, limiting confidence in assay reproducibility. Most studies relied on spiked samples with limited benchmarking against commercial or gold-standard methods, underscoring the need for standardized validation, rigorous comparative studies, and testing in naturally contaminated or clinical samples to support translational adoption.

## Figures and Tables

**Table 1:** Immunological and structural advantages of VHHs compared to conventional antibodies.

Features	VHH	Antibodies	Ref
CDR3 epitope reach	Elongated and flexible; access to recessed/cryptic epitopes	shorter; epitope access sometimes sterically limited recessed or hidden epitopes; increases paratope diversity	[32,33,34]
Fc region/effector functions	Absent: lack Fc effector functions; are not bound by SpG and only sporadically bound by SpA.	Present: enable Fc-driven effects; bind Prot A/G; hinge can be protease-cleaved	[35]
Engineering flexibility	Easily engineered into homo/heterodimers or multimers via Fc fusion or simple tandem linkage	IgG engineering is possible but complicated	[36]
Thermostability	High thermostability	Limited thermostability	[37,38]
Production/expression systems	High-yield, low-cost microbial expression; no glycosylation required; easy engineering	Require mammalian cell systems; expensive and time-consuming; glycosylation needed for proper function	[39,40]
Molecular architecture	Single VHH domain	IgG heterotetramer of two heavy and two light chains with multiple domains (VL, CL; VH, CH1-CH3), organized into Fab and Fc regions	[32]

**Table 2:** Summary of nanobody-based immunoassays for bacterial pathogen detection in food and clinical samples. Abv: LFIA (lateral flow immunoassay), ITS (immunochromatographic test strip), AuNP (gold nanoparticles), ELASA (sandwich-enzyme-linked aptamer sorbent assay), C-ELISA (dual-mode immunoassay colorimetric ELISA), P-ELISA (phage-mediated sandwich ELISA), IMS-ELISA (immunomagnetic separation ELISA), diVHH (dimeric variable domain of a heavy-chain-only antibody), AuSPE (screen-printed gold electrodes), TAS-ELISA (triple-antibody sandwich ELISA).

Spiked Samples	Detection Method	Target Pathogen	Capture Ab/Nb	Detection Ab/Nb/Probe	LOD	Ref
Beef, milk, orange juice	Nb-based double sandwich ELISA	*E. coli* O157:H7	Polyclonal rabbit anti-*E.coli* O157:H7	Nb O3	8.7 × 10^3^ cfu/mL	[45]
Milk	Nb-based sandwich ELISA	*S. enteritidis*	Polyclonal rabbit anti-*S. enteritidis*	Nb13	Direct assay: LOD = 1.4 × 10^5^ CFU/mL After enrichment (10 h): LOD improved to 6 CFU/mL	[46]
Pasteurized milk	Nb-based sandwich ELISA	*L. monocytogenes*	MAb 4A7	Nanobody L5-79	1 × 10^4^ CFU/mL in milk	[47]
Ham sausage, beef, shrimp	Sandwich ELISA based on bivalent nanobody capture and phage-displayed nanobody detection	*S. enteritidis* and *serotypes*	Bivalent VHH (Nb422-422)	Nb422+ HRP-conjugated secondary antibody	2.36 × 10^3^ CFU/mL (7.5-fold improvement over conventional nanobody ELISA) conventional monovalent nanobody-based ELISA LOD ~1.8 × 10^4^ CFU/mL	[48]
Milk, juice, pork	LFIA with streptavidin–biotin oriented AuNP conjugates (“Molecular Velcro”)	*S. typhimurium*	Rabbit polyclonal antibody (immobilized on test line)	Biotinylated nanobody (Nb9) conjugated with streptavidin-coated AuNPs (Au/SA@Bio-Nb)	Visual detection limit = 10^3^ CFU/mL	[49]
Milk, honey, pork, lettuce	Streptavidin-bridged self-paired sandwich ELISA	*S. typhimurium*, *S. enteritidis*, *S. london*, *S. paratyphi* B, *S. hadar*	Biotinylated nanobody (Nb-01) immobilized via streptavidin	Nb-01 + HRP-conjugated anti-M13 antibody	Conventional Nb sandwich ELISA (passive adsorption): LOD ~2.6 × 10^4^ CFU/mL (lowest sensitivity) SAB-ELISA (biotin–streptavidin Nb capture): LOD 4.23–9.15 × 10^3^ CFU/mL (6-fold improvement); enhanced SAB-ELISA (streptavidin bridging + phage amplification): best performance, LOD consistently ~4–9 × 10^3^ CFU/mL	[50]
Juice, honey, chocolate	ITS with colorimetric (AuNP signal) and photothermal (KMO@Au nanoflower + infrared laser readout) detection	*S. typhimurium*	Polyclonal antibody anti-*S. typhimurium* immobilized on test line + anti-HA tag antibody on control line	Nb9 conjugated to KMO@Au photothermal probes	Colorimetric mode: 1 × 10^4^ CFU/mL Photothermal mode: 1 × 10^3^ CFU/mL (10-fold better than colorimetric)	[51]
Clinical isolates	ELASA	*A. baumannii*	Nb anti-Bap protein	Biotinylated aptamer Aci49	1 × 10^3^ CFU/mL	[43]
Juice, honey, milk, and pork	Nb-based ELISA P-ELISA P-CLISA	*S. typhimurium*	Nb1 anti-*S. typhimurium*	Nb-ELISA: Soluble Nb-HRP fusion P-ELISA: Phage-displayed Nb + HRP-conjugated anti-M13 antibody P-CLISA: Phage-displayed Nb + HRP-conjugated anti-M13 antibody + chemiluminescent substrate	Nb-ELISA: 5.08 × 10^6^ CFU/mL (lowest sensitivity) P-ELISA (phage-mediated): 5.08 × 10^4^ CFU/mL (~100-fold better than Nb-ELISA due to phage signal amplification) P-CLISA (chemiluminescent format): 3.63 × 10^3^ CFU/mL (~14-fold better than P-ELISA). Linear range: 5.1 × 10^3^–1.2 × 10^6^ CFU/mL 14-fold more sensitive than P-ELISA	[52]
Chicken meat, cabbage, tomato, apple juice	Nb-based sandwich ELISA coupled with IMS-ELISA	*S. enteritidis*	Soluble Nb-F18	Nb Phage-F23 (specific for O antigen)	IMS-ELISA LOD: 3.2 × 10^3^ CFU/mL In spiked food samples (LOD50 = 6.9 × 10^3^ CFU/25 g or mL, LOD95 = 3.0 × 10^4^ CFU/25 g or mL)	[53]
Lettuce, chicken, pork	diVHH ELISA diVHH-based sandwich ELISA	*S. typhimurium*	diVHH-I	Biotinylated VHH-II	Biotinylated diVHH ELISA: 1.80 × 10^6^ CFU/mL (activated cells), 1.51 × 10^5^ CFU/mL (inactivated cells) Sandwich ELISA: 1.04×10^2^ CFU/mL (inactivated *S. typhimurium*) After 6 h enrichment in lettuce: ~10 CFU/mL detectable	[54]
Shrimp	One-step, label-free colorimetric biosensor using thiolated phage-displayed nanobody (Phage-Nb-SH) + AuNP aggregation	*V. parahaemolyticus*	Phage-Nb20-SH acts as capture and recognition element	Phage-Nb20-SH anchored to gold nanoparticles (AuNPs)	Visual detection limit: 1 × 10^4^ CFU/mL (naked eye) Quantitative detection limit: 1 × 10^3^ CFU/mL (UV–vis spectrometry)	[55]
Milk	Sandwich ELISA	*S. aureus*	His-tagged Nb147	Biotinylated Nb147	1.4 × 10^5^ CFU/mL (*S. aureus*) in PBS	[56]
Milk, pork	Fe_3_O_4_@SiO_2_@TQD dual-probe LFIA	*S. enteritidis*, *L. monocytogenes*, *C. jejuni*	mAbs (specific to SE, LM, CJ) immobilized on test lines.	Nanobody–mAb probes coupled with Fe_3_O_4_@SiO_2_@TQD (triple-quantum dot) nanoparticles	Dual-probe LFIA: SE 260 CFU/mL, LM 674 CFU/mL, CJ 264 CFU/mL AuNP-based LFIA (comparator): SE 1 × 10^5^ CFU/mL, LM 1 × 10^5^ CFU/mL, CJ 3 × 10^4^ CFU/mL Dual-mAb ELISA (comparator): SE 2427 CFU/mL, LM 14,267 CFU/mL, CJ 9154 CFU/mL	[57]
Saliva-like biological fluids	Electrochemical immunosensor using AuSPE Detection via voltammetric techniques (CV, DPV, SWV)	*H. pylori*	Immobilized nanobody D2_Nb on electrode surface	Not a sandwich assay, antigen detection monitored by electrochemical signal variation CV (Cyclic Voltammetry), DPV (Differential Pulse Voltammetry), SWV (Square Wave Voltammetry), EIS (Electrochemical Impedance Spectroscopy)	CV: 7.2 ng/mL DPV: 4.9 ng/mL SWV: 3.1 ng/mL EIS: 3.4 ng/mL	[58]
Lettuce, meat	Colorimetric nanozyme assay (β-CD@AuNP peroxidase-like activity)	*S. typhimurium*	VHHs conjugated to AuNPs catalytic/color signal generator	VHH-AuNP conjugates used for both binding and colorimetric signal generation	reported equivalent to ~10^2^–10^3^ CFU/mL	[59]
Fecal samples from camel calves	Magnetofluorescent nanobody-based sandwich assay	*E. coli* F17 strains	Nb1 anti-F17A, conjugated to MBs	Nb4 conjugated to horseradish peroxidase (HRP), producing fluorescent signal with OPD/H_2_O_2_ substrate	1.8 CFU/mL for *E. coli* F17, using the magnetofluorescent nanobody-based assay significantly more sensitive than conventional ELISA: LOD of 10^4^ CFU/mL	[60]
Milk	TAS-ELISA	*E. coli* O157:H7	mAb specific for *E. coli* O157:H7	Nb-4-E-10 against EspA (A89-I119) and Nb-4-O-3 against OmpA (K294-Q316))	mAb-ELISA (control): 7.9 × 10^4^ CFU/mL DAS1-ELISA: 1.72 × 10^4^ CFU/mL DAS2-ELISA: 2.63 × 10^4^ CFU/mL TAS-ELISA: 1.89 × 10^3^ CFU/mL	[61]
Skimmed milk, chicken intestinal tract, and organs	Double Nb-based sandwich ELISA for the detection of *S. enteritidis* in milk and in vivo for chicken organs	*S. enteritidis* serovar	Nanobody (specific to *S. enteritidis* O/H antigens)	Nanobody (paired, self-paired sandwich format, HRP-conjugated)	5 × 10^4^ CFU/mL	[62]

**Table 3:** Summary of nanobody-based immunoassays for bacterial toxin detection in food and clinical samples. Abv: RANbody (reporter–nanobody fusions), C-ELISA (dual-mode immunoassay colorimetric ELISA), F-ELISA (fluorescent ELISA), CLIA (chemiluminescent immunoassay), NLFIA (nanobody-armed photothermal lateral flow immunoassay), TRFICA (time-resolved fluorescence immunochromatographic assay), EIS (electrochemichal impedance spectroscopy immunosensor).

Spiked Samples	Detection Method	Target Pathogen	Capture Ab/Nb	Detection Ab/Nb/Probe	LOD	Ref
Milk and pork	Double-Nb sandwich ELISA with nanobody–HRP fusion (RANbody)	*S. aureus* α-hemolysin (Hla) toxin	Nb HLA39	Nb HLA17 fused with HRP (RANbody)	10 ng/mL for α-hemolysin	[63]
Milk and pork	CELISA FELISA	SEA	Nb SEA18	Nb SEA33-vHRP	CELISA: 0.09 ng/mL FELISA: 0.40 ng/mL (~16× lower than traditional antibody-based ELISA)	[64]
Milk, drinking water, human serum	CLIA	SEB	Anti-SEB mAb	Nb37–Alkaline ALP fusion protein	LOD: 1.44 ng/mL	[65]
Milk, milk powder, pork	Sandwich ELISA (mAbs-ELISA, VHH-ELISA, OtTNb ELISA).	SEA	Soluble Nb26	Phage-displayed Nb12 + Nb150 (dual reporters)	mAbs-ELISA: 1.47 ng/mL VHH-ELISA (single phage Nb): 0.80–0.89 ng/mL OtTNb ELISA (dual-phage Nb12 + Nb150): 0.43 ng/mL	[66]
Milk, milk powder, pork	NLFIA	SEB	Anti-SEB Nb7 immobilized on test line	Au core–petal nanoparticle (CPN)-labeled Nb7	Traditional AuNPs-LFIA: 14.8 ng/mL NLFIA (colorimetric readout): 1.68 ng/mL NLFIA (photothermal readout): 0.58 ng/mL ~25-fold sensitivity improvement compared to conventional AuNPs-LFIA	[67]
Milk, pork	Dual-mode nanobody immunoassay (colorimetric + fluorescence) using a bifunctional protein RANbody	SEB	SEB27-vHRP	SEB57 Nb	0.12 ng/mL (colorimetric); 0.24 ng/mL (fluorescence)	[68]
Controlled laboratory samples (Luminex-bead-based system)	Luminex-based multiplex bead assay (sandwich immunoassay)	SEB	Anti-SEB Nb (sdAb A3), llama polyclonal IgG1/IgG2, (MAb 3b2a)	Biotinylated sdAb A3, biotinylated llama IgG, biotinylated MAb 3b2a	sdAb A3 as capture + biotinylated MAb 3b2a detector: LOD ~64 pg/mL SEB; reverse (MAb capture + A3 detector): LOD ~40 ng/mL sdAb A3 alone as capture/detector: detection down to 64 pg/mL	[69]
Milk, sand extract solution, healthy human serum	ELISA 1: Streptavidin-bridged double-nanobody ELISA ELISA 2: Nanobody–HRP direct fusion ELISA TRFICA: Using lanthanide nanospheres.	BoNT/A and BoNT/B	VHH BoNT/A and Nb BoNT/B	ELISA 1: Nanobody tracers + streptavidin–HRP. ELISA 2: Nanobody directly fused with HRP (self-reporting). TRFICA: Nanobody conjugated to lanthanide nanospheres (fluorescent signal generator)	ELISA 1: 0.17 ng/mL (BoNT/A in milk), 0.3 ng/mL (BoNT/A in human serum) ELISA 2: ~4.5–15 ng/mL (depending on matrix) TRFICA: 0.05 ng/mL (similar to mouse bioassay gold standard)	[44]
Milk, yogurt, cheese	VHH sandwich ELISA	SEC	Anti-SEC Nb C6	Phage-displayed anti-SEC nanobody C11 (with HRP-conjugated anti-M13 antibody as signal amplifier	LOD: 2.47 ng/mL	[70]
Whole milk and 2% milk	Sandwich ELISA and direct ELISA	SEB	SEB-12 Nb Other VHHs tested: SEB-6, SEB-18, SEB-20, SEB-62 paired with pAb	pAb	SEB-12 nanobody + polyclonal antibody ELISA (best pair): LOD in PBS: 0.19 ng/mL LOD in whole milk: 0.39 ng/mL LOD in 2% milk: 0.39 ng/mL Nanobody–pAb combinations: LODs ranged between 0.19–0.39 ng/mL	[71]
Stool sample	Double-VHH sandwich ELISA	Stx2	1vb1	biotinylated 2vb10	LOD in ELISA (1vb1–2vb10, biotinylated detection): 9.2 pg/mL of purified Stx2a (buffer) LOD in stool-spiked samples: 10–12 pg/mL 4× lower than the commercial R-Biopharm ELISA kit, which had an LOD of ~40 pg/mL under the same conditions	[72]
Feces/cecal samples	Sandwich ELISA-based quantification assays	TcdA and TcdB	TcdA: A2B10 (anti-CROPs) A1D8 (anti-DD) TcdB: B2C11 (anti-GTD) B0D10 (anti-DD)	TcdA: A1A6 (anti-GTD) A1C3 (anti-DD) TcdB: B0E2 (anti-DD) B0E2 (anti-DD)	TcdA (A2B10/A1A6 pair): LOD: 0.6 ng/mL in buffer/culture supernatant LOD: 12 ng/mL in feces/cecal content TcdA (A1D8/A1C3 pair): LOD: 0.019 ng/mL in buffer, 0.075 ng/mL in feces, 0.6 ng/mL in cecal content TcdB (B2C11/B0E2 pair): LOD: 2.1 ng/mL in buffer and culture supernatant TcdB (B0D10/B0E2 pair): LOD: 0.033 ng/mL in buffer, 0.65 ng/mL in culture supernatant	[73]
Stool sample	Sandwich-type EIS with gold electrode modified by cystamine SAM, primary sdAb immobilization, toxin binding, and secondary sdAb-coated AuNPs for signal amplification.	TcdA and TcdB	sdAb1	sdAb2–AuNP	TcdA: 0.61 pg/mL (S/N = 3). TcdB: 0.60 pg/mL (S/N = 3).	[74]

**Table 4:** Adaptation of the QUADAS-2 tool for nanobody-based immunoassays. This table presents the adapted QUADAS-2 framework applied to the evaluation of nanobody-based immunoassays designed for detecting bacterial pathogens and toxins in various matrices, including food and human samples. In this adaptation, the original “patient selection” domain has been reformulated as “sample selection” to reflect the nature of diagnostic accuracy assessments outside of clinical settings.

Domain	Adaptation	Typical Outcome Across 32 Studies
D1: Sample Selection	Replaces “patient selection.” Refers to whether studies used consecutive/random food/clinical/environmental samples or only spiked samples.	Some concerns in most studies: almost all used spiked samples, not naturally contaminated or consecutive field samples. Applicability concern: moderate–high (samples not fully representative).
D2: Index Test (Nanobody Assay)	Whether assays were interpreted blinded to the reference standard, thresholds were pre-specified.	Low for most: index tests well-described, pre-specified LODs, cutoffs. Applicability: low (nanobody assays directly matched the review question).
D3: Reference Standard	Culture, PCR, or commercial ELISA used as gold standards.	Low in most: standards well-established and appropriate. In a few, no comparator (risk: high). Applicability: low (reference methods aligned with diagnostic question).
D4: Flow and Timing	Timing between the nanobody assay and reference standard.	Low to some concerns: Most studies tested all prepared samples with immediate timing.

## Data Availability

The original contributions presented in this study are included in the article/Supplementary Material. Further inquiries can be directed to the corresponding authors.

## Supplementary Materials

The following supporting information can be downloaded at: https://www.mdpi.com/article/10.3390/antib15010015/s1, Table S1: Research equation and relevant Keywords used to conduct the search; Table S2: Summary of Nanobody-based immunoassays for bacteria and toxin detection in food and clinical samples. Abv: LFIA (lateral flow immunoassay), ITS (immunochromatographic test strip), AuNP (gold nanoparticules), ELASA (Sandwich enzyme-linked aptamer sorbent assay), C-ELISA (dual mode immunoassay colorimetric ELISA), P-ELISA (phage mediated sandwich ELISA), IMS-ELISA (immunomagnetic separation ELISA), diVHH (dimeric variable domain of a heavy-chain-only antibody), AuSPE (screen printed gold electrodes), TAS-ELISA (triple antibody sandwich ELISA), RANbody (Reporter-nanobody fusions), C-ELISA (dual mode immunoassay colorimetric ELISA), F-ELISA (fluorescent ELISA), CLIA (chemiluminescent immunoassay), NLFIA (nanobody-armed photothermal lateral flow immunoassay), TRFICA (time-resolved fluorescence immunochromatographic assay), EIS (electrochemichal impedance spectroscopy immunosensor); Table S3: Summary of the main detection methods evaluated across 32 studies selected, according to their sensitivity, specificity, and overall practicality in detection applications.
