# Supplementary material for: Analytical Performance of Nanobody-Based Immunoassay and Immunosensing Platforms for Bacteria and Toxin Detection: A Systematic Review
Source: Antibodies (Basel). 2026 Feb 21;15(1):15. doi: 10.3390/antib15010015 (PMC12937825; doi:10.3390/antib15010015)
Supplement: Supplementary file 1 [file antibodies-15-00015-s001.zip › antibodies-4016512-supplementary.pdf]

Supplementary material

MATERIALS AND METHODS

1. Search strategy

Data base	Research equation
**Pubmed**	("Nanobodies"[Mesh] OR "Single-Domain Antibodies"[Mesh] OR "Camelid Antibodies"[Mesh] OR "VHH Antibodies"[Mesh] OR nanobody[Title/Abstract] OR nanobodies[Title/Abstract] OR "single-domain antibody"[Title/Abstract] OR VHH[Title/Abstract] OR "camelid antibody"[Title/Abstract]) AND ("Bacterial Infections"[Mesh] OR "Bacteria"[Mesh] OR bacteria[Title/Abstract] OR "bacterial toxin"[Mesh] OR "bacterial toxins"[Title/Abstract] OR toxin[Title/Abstract] OR toxins[Title/Abstract] OR "virulence factor"[Title/Abstract] OR "enterotoxin"[Title/Abstract]) AND ("Immunoassay"[Mesh] OR immunoassay[Title/Abstract] OR ELISA[Title/Abstract] OR "lateral flow"[Title/Abstract] OR LFIA[Title/Abstract] OR biosensor*[Title/Abstract] OR "electrochemical sensor"[Title/Abstract] OR "colorimetric assay"[Title/Abstract])
**Scopus**	TITLE-ABS-KEY("nanobody" OR "nanobodies" OR "single-domain antibody" OR "single-domain antibodies" OR "VHH") AND TITLE-ABS-KEY("bacterial infection" OR "bacteria" OR "bacterial toxin") AND TITLE-ABS-KEY("immunoassay" OR "biosensor" OR "biosensing technique" OR "detection platform")
**PMC**	("Single-Domain Antibodies"[Mesh] OR "Nanobody"[Title] OR "Nanobodies"[Title] OR "Single-Domain Antibody"[Title] OR "VHH"[Title] OR "Camelid Antibody"[Title]) AND ("Bacterial Infections"[Mesh] OR "Bacteria"[Mesh] OR "Bacterial Toxins"[Mesh]) AND ("Immunoassay"[Mesh] OR "Biosensing Techniques"[Mesh])
**Sciencedirect**	nanobody-based immunoassays

Table S1 Research equation and relevant eywords used to conduct the search

RESULTS AND DISCUSSION

1. Overview of nanobody-based immunoassays: technical aspects, performance, and practicality

Spiked Samples	Detection method	Target Pathogen/Toxin	Capture Ab/Nb	Detection Ab/Nb/Probe	LOD	Recovery (Matrix Effect)	ref
**Beef, milk, orange juice**	Nb-based double sandwich ELISA	E.coli O157:H7	Polyclonal rabbit anti-E.coli O157:H7	Nb O3 (selected for high affinity and heat stability)	8.7 × 10^3 cfu/mL	Milk: 94–141% (improved to 73–104% after 1:100 dilution). Enrichment broth: 144–220% (improved to 97–118% after dilution)	(19)
**milk**	Nb-based sandwich ELISA	S.enteritidis	Polyclonal rabbit anti- S. enteritidis	Nb13 (selected for high affinity, thermostability, specificity)	Direct assay (no enrichment): LOD = 1.4 × 10^5 CFU/mL<br>After enrichment (10 h): LOD improved to 6 CFU/mL	70–95%	(20)
**pasteurized milk**	Nb-based sandwich ELISA	L.monocytogenes	Monoclonal antibody (MAb 4A7)	Nanobody L5-79 (derived from camelid VHH library)	1 × 10^4 CFU/mL in milk.	84–96%	(21)
**Milk and pork**	Double-Nb sandwich ELISA with nanobody-HRP fusion (RANbody)	S.aureus α-hemolysin (Hla) toxin	Nanobody HLA39	Nanobody HLA17 fused with HRP (RANbody), eliminating the need for secondary antibodies	10 ng/mL for α-hemolysin	Milk: 94.5%–108%<br>Pork: 90.8%–97.6%	(22)
**Ham sausage, beef, shrimp**	Sandwich ELISA based on bivalent nanobody capture and phage-displayed nanobody detection	S.enteritidis and serotypes	Bivalent nanobodies (Nb422-422)	Phage-displayed nanobody Nb422+ HRP-conjugated secondary antibody	2.36 × 10^3 CFU/mL (7.5-fold improvement over conventional nanobody ELISA) conventional monovalent nanobody-based ELISA (LOD ~1.8 × 10^4 CFU/mL)	Ham sausage, beef, shrimp: 73%–125.6%	(23)
**Milk, juice, pork**	LFIA with streptavidin-biotin oriented AuNP conjugates (“Molecular Velcro”)	S.typhimurium	Rabbit polyclonal antibody (immobilized on test line)	Biotinylated nanobody (Nb9) conjugated with streptavidin-coated AuNPs (Au/SA@Bio-Nb)	Visual detection limit = 10^3 CFU/mL	Milk: 84.8–93.7%<br>Juice: 89.3–98.1%<br>Pork: 86.7–95.0%	(24)

						Overall recoveries: 81.2–105.0%	
**Milk, honey, pork, lettuce**	Streptavidin-bridged self-paired sandwich ELISA	S.Typhimurium, S. Enteritidis, S. London, S. Paratyphi B, S. Hadar	Biotinylated nanobody (Nb-01) immobilized via streptavidin	Phage-displayed nanobody (Nb-01) + HRP-conjugated anti-M13 antibody	Conventional Nb sandwich ELISA (passive adsorption): LOD ~2.6×10^4 CFU/mL (lowest sensitivity) SAB-ELISA (biotin-streptavidin Nb capture): LOD 4.23–9.15×10^3 CFU/mL (6-fold improvement) Enhanced SAB-ELISA (streptavidin bridging + phage amplification): Best performance, LOD consistently ~4–9×10^3 CFU/mL	86.9%–106.6%	(25)
**Juice, honey, chocolate**	Dual-mode immunochromatographic test strip (ITS) with colorimetric (AuNP signal) and photothermal (KMO@Au nanoflower + infrared laser readout) detection	S.Typhimurium	Polyclonal antibody against S. Typhimurium immobilized on test line + anti-HA tag antibody on control line	Nanobody Nb9 conjugated to KMO@Au photothermal probes	Colorimetric mode: 1×10^4 CFU/mL Photothermal mode: 1×10^3 CFU/mL (10-fold better than colorimetric)	79.4%–117.4% (colorimetric mode), 81.9%–115.1% (photothermal mode)	(26)
**Clinical isolates from hospital**	Sandwich Enzyme-Linked Aptamer Sorbent Assay (ELASA)	A.baumannii	Nanobody (VHH) against Bap protein, coated on 96-well plates	Biotinylated aptamer Aci49 (selected via 12 rounds of whole-cell SELEX, Kd = 7.5 ± 1.35 pM, binding efficiency ~47.5%)	1×10^3 CFU/mL (*A. baumannii*)	ND	(18)

**pork and milk**	Dual-mode immunoassay: Colorimetric ELISA (CELISA) + Fluorescent ELISA (FELISA)	S.aureus enterotoxin A (SEA)	Nanobody SEA18 (selected from immune phage display)	Nanobody SEA33 fused with HRP to form a bifunctional protein (SEA33-vHRP)	CELISA: 0.09 ng/mL<br>FELISA: 0.40 ng/mL<br>(~16× lower than traditional antibody-based ELISA)	93.63–104.96%	(27)
**juice, honey, milk, and pork**	Nb-ELISA (conventional sandwich ELISA with nanobody)<br>P-ELISA (phage-mediated sandwich ELISA, phage-displayed nanobody as detection probe)<br>P-CLISA (phage-mediated chemiluminescent ELISA with nanobody capture + phage nanobody detection + HRP chemiluminescence readout)	S.enterica serovar Typhimuriu m	Nanobody Nb1 against S. Typhimurium (immobilized on plates)	Nb-ELISA: Soluble Nb-HRP fusion<br>P-ELISA: Phage-displayed Nb + HRP-conjugated anti-M13 antibody<br>P-CLISA: Phage-displayed Nb + HRP-conjugated anti-M13 antibody + chemiluminescent substrate	Nb-ELISA: 5.08×10^6 CFU/mL (lowest sensitivity)<br>P-ELISA (phage-mediated): 5.08×10^4 CFU/mL (~100-fold better than Nb-ELISA due to phage signal amplification)<br>P-CLISA (chemiluminescent format): 3.63×10^3 CFU/mL (~14-fold better than P-ELISA). Linear range: 5.1×10^3 – 1.2×10^6 CFU/mL 14-fold more sensitive than P-ELISA	88.6–108.2%	(28)
**Pure milk, drinking water, human serum**	Sandwich chemiluminescent immunoassay (CLIA)	S.enterotoxi n B (SEB)	Anti-SEB monoclonal antibody (mAb)	Nb37–Alkaline phosphatase (ALP) fusion protein	LOD: 1.44 ng/mL	Pure milk: 83.8–95.2%<br>Drinking water: 86.4–93.9%<br>Human serum: 82.5–88.5%	(29)
**milk, milk powder, pork**	Sandwich ELISA (three formats compared: mAbs-ELISA, Nbs-ELISA, OtTNb ELISA).	S.enterotoxi n A (SEA)	Soluble Nb26 (nanobody)	Phage-displayed Nb12 + Nb150 (dual reporters)	mAbs-ELISA: 1.47 ng/mL<br>Nbs-ELISA (single phage Nb): 0.80–0.89 ng/mL	Milk: 79.2–108.7%<br>Milk powder: 77.5–103.1%<br>Pork: 80.5–125.4%	(30)

					OtTNb ELISA (dual phage Nb12 + Nb150): 0.43 ng/mL		
**Chicken meat, cabbage, tomato, apple juice**	Nanobody-based sandwich ELISA coupled with immunomagnetic separation (IMS-ELISA)	S.enteritidis	Soluble nanobody Nb-F18 (specific for S. enteritidis H antigen)	Phage-displayed nanobody Phage-F23 (specific for O antigen)	IMS-ELISA LOD: 3.2×10^3 CFU/mL. In spiked food samples (LOD50 = 6.9 $\times 10^3$ CFU/25 g or mL, LOD95 = 3.0 $\times 10^4$ CFU/25 g or mL).	Chicken: 87–96%<br>Cabbage: 82–117%<br>Tomato: 88–97%<br>Apple juice: 93–116%	(31)
**Lettuce, chicken, pork**	ELISA (two types developed: biotinylated diVHH ELISA and diVHH-based sandwich ELISA)	S.Typhimurium	diVHH (divalent VHH-I)	Biotinylated VHH-II (for sandwich ELISA)	Biotinylated diVHH ELISA: 1.80×10^6 CFU/mL (activated cells), 1.51×10^5 CFU/mL (inactivated cells).<br>Sandwich ELISA: 1.04×10^2 CFU/mL (inactivated S. Typhimurium).<br>After 6 h enrichment in lettuce: ~10 CFU/mL detectable.	lettuce: 92–100%<br>chicken: 83–104%<br>pork: 96–106%	(32)
**Milk, milk powder, pork**	Nanobody-armed photothermal lateral flow immunoassay (NLFIA)	S.enterotoxin B (SEB)	Anti-SEB nanobody (Nb7) immobilized on test line	Au core–petal nanoparticle (CPN)-labeled Nb7 nanobody	Traditional AuNPs-LFIA: 14.8 ng/mL.<br>NLFIA (colorimetric readout): 1.68 ng/mL.<br>NLFIA (photothermal readout): 0.58 ng/mL.<br>~25-fold sensitivity improvement compared to conventional AuNPs-LFIA.	Milk: 89.4–112%<br>Milk powder: 87.2–105%<br>Pork: 90.3–108%	(33)
**Shrimp**	One-step, label-free colorimetric biosensor using thiolated phage-displayed	V.parahaemolyticus	(Phage-Nb20-SH) The nanobody itself acts as both	Detection reagent: A thiolated phage-displayed nanobody (Phage-Nb20-SH) anchored to	Visual detection limit: 1×10^4 CFU/mL (naked eye).	97.9–104.5%	(34)

	nanobody (Phage-Nb-SH) + AuNP aggregation		capture and recognition element	gold nanoparticles (AuNPs) specifically bind Vibrio parahaemolyticus induces visible color change (red → purple/blue). The nanobody itself acts as both capture and recognition element	Quantitative detection limit: 1 × 10^3 CFU/mL (UV–vis spectrometry)		
**Milk, pork**	Dual-mode nanobody immunoassay (colorimetric + fluorescence)	S.enterotoxin B (SEB)	nanobody (anti-SEB VHH) immobilized on plate provided high specificity and avoided interference from Staphylococcal protein A (SpA), which often binds nonspecifically to IgG in conventional assay	nanobody–HRP fusion protein (RANbody) providing dual colorimetric & fluorescent signals.	0.12 ng/mL (colorimetric); 0.24 ng/mL (fluorescence)	Milk: 95–110%<br>Pork: 96–107%	(35)
**Milk**	Sandwich ELISA	S.aureus	His-tagged Nb147	Biotinylated Nb147	1.4 × 10^5 CFU/mL (S. aureus) in PBS	ND	(36)
**Milk, pork**	Fe_3O_4@SiO_2@TQD dual-probe LFIA (lateral flow immunoassay) with nanobody–mAb probe combinations and test lines coated with monoclonal antibodies.	S.enteritidis, L.monocytogenes, C.jejuni	Monoclonal antibodies (specific to SE, LM, CJ) immobilized on test lines.	Nanobody–mAb probes coupled with Fe_3O_4@SiO_2@TQD (triple-quantum dot) nanoparticles	Dual-probe LFIA: SE 260 CFU/mL, LM 674 CFU/mL, CJ 264 CFU/mL.<br>AuNP-based LFIA (comparator): SE 1 × 10^5 CFU/mL, LM 1 × 10^5 CFU/mL, CJ 3 × 10^4 CFU/mL.<br>Dual-mAb ELISA (comparator): SE 2427 CFU/mL, LM 14,267 CFU/mL, CJ 9154 CFU/mL.	Milk: 98.8–108.1%<br>Pork: 87.3–97.5%	(37)

**controlled laboratory samples (Luminex bead-based system)**	Luminex-based multiplex bead assay (sandwich immunoassay)	S.aureus enterotoxin B (SEB)	Anti-SEB nanobody (sdAb A3), llama polyclonal IgG1/IgG2, monoclonal antibody (MAb 3b2a)	Biotinylated sdAb A3, biotinylated llama IgG, biotinylated MAb 3b2a.	sdAb A3 as capture + biotinylated MAb 3b2a detector: LOD ~64 pg/mL SEB.<br>Reverse (MAb capture + A3 detector): LOD ~40 ng/mL.<br>sdAb A3 alone as capture/detector: detection down to 64 pg/mL.	ND	(38)
**saliva-like biological fluids**	Electrochemical immunosensor using screen-printed gold electrodes (AuSPE). Detection via voltammetric techniques (CV, DPV, SWV)	H.pylori	Immobilized nanobody D2_Nb on electrode surface	Not a sandwich assay — antigen detection monitored by electrochemical signal variation CV (Cyclic Voltammetry), DPV (Differential Pulse Voltammetry), SWV (Square Wave Voltammetry),<br>EIS (Electrochemical Impedance Spectroscopy)	CV: 7.2 ng/mL<br>DPV: 4.9 ng/mL<br>SWV: 3.1 ng/mL<br>EIS: 3.4 ng/mL	Artificial Saliva: 91.9% (10 ng/mL), 103.4% (100 ng/mL), 96.2% (1000 ng/mL)	(39)
**lettuce, meat extracts**	Colorimetric nanozyme assay (β-CD@AuNP peroxidase-like activity)	S.Typhimurium	VHHs conjugated to AuNPs (VHH = recognition/specificity (binds Salmonella)<br>AuNP = catalytic/color signal generator	VHH-AuNP conjugates used for both binding and colorimetric signal generation	Achieved in the ng/mL range (reported equivalent to ~10^2–10^3 CFU/mL)	80–105%	(40)
**milk, Sand extract solution,**	ELISA 1: Streptavidin-bridged double nanobody ELISA.<br>ELISA 2: Nanobody–HRP	BoNT/A and BoNT/B	VHHs specific to BoNT/A and BoNT/B (A-capture and B-capture nanobodies)	ELISA 1: Nanobody tracers + streptavidin–HRP.<br>ELISA 2: Nanobody directly fused with HRP (self-reporting).	ELISA 1: 0.17 ng/mL (BoNT/A in milk), 0.3 ng/mL (BoNT/A in human serum).<br>ELISA 2: ~4.5–15 ng/mL (depending	ELISA: 87–115% in milk, serum, and sand.<br>TRFICA: 85–149% across matrices.	(17)

**Healthy human serum**	direct fusion ELISA (avoids secondary antibody). TRFICA: Time-resolved fluorescence immunochromatographic assay using lanthanide nanospheres.			TRFICA: Nanobody conjugated to lanthanide nanospheres (fluorescent signal generator).	on matrix). TRFICA: 0.05 ng/mL (similar to mouse bioassay gold standard)		
**Bacterial cultures (E. coli F17, control strains). fecal samples from camel calves**	Magnetofluorescent nanobody-based sandwich assay	E. coli F17 strains	Nb1 (anti-F17A), conjugated to MBs	Nb4 conjugated to horseradish peroxidase (HRP), producing fluorescent signal with OPD/H_2O_2 substrate	1.8 CFU/mL for E. coli F17, using the magnetofluorescent nanobody-based assay. significantly more sensitive than conventional ELISA: LOD of 10^4 CFU/mL	89–102%	(41)
**milk**	Phage-mediated triple antibody sandwich immunoassay (TAS-ELISA)	E.coli O157:H7	monoclonal antibody (mAb) specific for E. coli O157:H7	two phage-displayed nanobodies (Nb-4-E-10 against EspA (A89-I119) and Nb-4-O-3 against OmpA (K294-Q316))	mAb-ELISA (control): 7.9×10^4 CFU/mL<br>DAS1-ELISA: 1.72×10^4 CFU/mL<br>DAS2-ELISA: 2.63×10^4 CFU/mL<br>TAS-ELISA: 1.89×10^3 CFU/mL	TAS-ELISA recoveries: 83.3% – 90.7% (intra-assay), 76.3% – 105.2% (inter-assay).	(42)
**milk, yogurt, cheese**	Nanobody-based sandwich ELISA (Nbs-ELISA)	S.enterotoxin C (SEC)	Anti-SEC nanobody C6	Phage-displayed anti-SEC nanobody C11 (with HRP-	LOD: 2.47 ng/mL	84.5–108%.	(43)

				conjugated anti-M13 antibody as signal amplifier			
**Whole milk and 2% milk**	Sandwich ELISA and direct ELISA	S.enterotoxin B (SEB)	Best-performing: SEB-12 nanobody (capture)<br>Other Nbs tested: SEB-6, SEB-18, SEB-20, SEB-62 nanobodies paired with pAb (moderate performance).	Polyclonal antibody (pAb)	SEB-12 nanobody + polyclonal antibody ELISA (best pair):<br>LOD in PBS: 0.19 ng/mL.<br>LOD in whole milk: 0.39 ng/mL.<br>LOD in 2% milk: 0.39 ng/mL.<br>Nanobody–pAb combinations: LODs ranged between 0.19–0.39 ng/mL	ND	(44)
**stool sample**	double-VHH sandwich ELISA for Stx2 detection	Shiga toxin2	1vb1	biotinylated 2vb10	LOD in ELISA (1vb1–2vb10, biotinylated detection): 9.2 pg/mL of purified Stx2a (buffer).<br>LOD in stool-spiked samples : 10–12 pg/mL 4× lower than the commercial R-Biopharm ELISA kit, which had an LOD of ~40 pg/mL under the same conditions.	95–105%	(45)
**feces/cecal samples**	sandwich ELISA-based quantification assays for TcdA and TcdB.	C. difficile TcdA and TcdB	TcdA: A2B10 (anti-CROPs) as capture<br>A1D8 (anti-DD) as capture<br>TcdB: B2C11 (anti-GTD) as capture<br>B0D10 (anti-DD) as capture	TcdA: A1A6 (anti-GTD) as detection<br>A1C3 (anti-DD) as detection (best-performing pair for complex samples) .<br>TcdB: B0E2 (anti-DD) as detection<br>B0E2 (anti-DD) as detection	TcdA (A2B10/A1A6 pair):<br>LOD: 0.6 ng/mL in buffer/culture supernatant .<br>LOD: 12 ng/mL in feces/cecal content .<br>TcdA (A1D8/A1C3 pair):<br>LOD: 0.019 ng/mL in buffer, 0.075 ng/mL in feces, 0.6 ng/mL in cecal content	ND	(46)

					TcdB (B2C11/B0E2 pair):<br>LOD: 2.1 ng/mL in buffer and culture supernatant .<br>TcdB (B0D10/B0E2 pair):<br>LOD: 0.033 ng/mL in buffer, 0.65 ng/mL in culture supernatant.		
**Skimmed milk, chicken intestinal tract, and organs**	Double nanobody-based sandwich ELISA (self-paired) for detection of S. Enteritidis in food (milk) and in vivo (chicken organs)	Salmonella enterica serovar Enteritidis (S. Enteritidis)	Nanobody (specific to S. Enteritidis O/H antigens)	Nanobody (paired, self-paired sandwich format, HRP-conjugated)	5 × 10^4 CFU/mL	97.02% – 108.59%	(47)
**stool sample**	Sandwich-type electrochemical impedance immunosensor (EIS) with gold electrode modified by cystamine SAM, primary sdAb immobilization, toxin binding, and secondary sdAb-coated AuNPs for signal amplification.	C.difficile toxins A (TcdA) and B (TcdB)	sdAb1 (single domain antibody, VHH) immobilized on gold electrode	sdAb2 (VHH) conjugated to AuNPs (sdAb2–AuNP) served as amplification probe	TcdA: 0.61 pg/mL (S/N = 3).<br>TcdB: 0.60 pg/mL (S/N = 3).	ND	(48)

Table S2 Summary of Nanobody-based immunoassays for bacteria and toxin detection in food and clinical samples. Abv: LFIA (lateral flow immunoassay), ITS (immunochromatographic test strip), AuNP (gold nanoparticules), ELASA (Sandwich enzyme-linked aptamer sorbent assay), C-ELISA (dual mode immunoassay colorimetric ELISA), P-ELISA (phage mediated sandwich ELISA), IMS-ELISA (immunomagnetic separation ELISA), diVHH (dimeric variable domain of a heavy-chain-only antibody), AuSPE (screen printed gold electrodes), TAS-ELISA (triple antibody sandwich ELISA), RANbody (Reporter-nanobody fusions), C-ELISA (dual mode immunoassay colorimetric ELISA), F-ELISA (fluorescent ELISA), CLIA (chemiluminescent immunoassay), NLFIA (nanobody-armed photothermal lateral flow immunoassay), TRFICA (time-resolved fluorescence immunochromatographic assay), EIS (electrochemichal impedance spectroscopy immunosensor)

Detection method	Comparator	Sensitivity	Specificity	practicality	ref
Nb-based double sandwich ELISA	NONE	Detects 5 cfu/25 mg (beef), 5 cfu/200 mL (milk, orange juice) after 5h enrichment	no cross-reactivity observed with other E. coli strains or pathogenic bacteria tested: Salmonella typhimurium, Listeria monocytogenes, Vibrio parahaemolyticus, and Staphylococcus aureus	Works in complex food matrices, rapid detection (<5h post-enrichment), high thermostability	(19)
Nb-based sandwich ELISA	NONE	Analytical sensitivity: LOD = 1.4×10^5 CFU/mL (direct), 6 CFU/mL (after 10h enrichment)	Nb13 and Nb16 recognized only S. enteritidis No cross-reactivity with 13 other tested pathogens (including Salmonella typhimurium, Listeria monocytogenes, Staphylococcus aureus, etc.)	Stability: Nanobodies retained ~90% activity after heating to 95 °C Reproducibility: Relative standard deviation (RSD) ~10.7% across replicates Ease of use: Standard ELISA format, compatible with food testing workflows	(20)
Nb-based sandwich ELISA	NONE	1×10^4 CFU/mL in milk.	Nanobodies L5-78 and L5-79 bound only to L. monocytogenes serotypes 1/2a, 1/2b, 4b. No cross-reactivity with seven other bacterial genera tested.	Nanobodies demonstrated thermostability and chemical stability, making them robust for food safety diagnostics. Recombinant expression in E. coli ensures low cost and scalability. The ELISA platform is compatible with existing food safety testing workflows.	(21)
Double-Nb sandwich ELISA with nanobody-HRP fusion (RANbody)	NONE	Linear detection range 10–1000 ng/mL, regression $R^2 = 0.998$	No cross-reactivity with β-hemolysin, γ-hemolysin, or S. aureus Δhla supernatant	Eliminates secondary antibodies (cost- and time-saving); simple recombinant production; stable and reproducible; high throughput potential	(22)
Sandwich ELISA based on bivalent nanobody capture and phage-displayed nanobody detection	Conventional nanobody-based ELISA	LOD 2.36×10^3 CFU/mL	High specificity: negligible cross-reactivity with non-Salmonella pathogens	timesaving, low-cost, and sensitive approach, demonstrated in spiked food matrices	(23)

LFIA with streptavidin-biotin oriented AuNP conjugates ("Molecular Velcro")	NONE	Good linearity (R^2 = 0.9955) between bacterial concentration and signal intensity; detectable at 1 CFU after enrichment (6–7 h in culture)	No cross-reactivity with S. enteritidis, S. hadar, S. london, Cronobacter sakazakii, Shigella flexneri, Staphylococcus aureus, or E. coli	Simple, rapid (15 min assay), portable, no professional equipment needed; improved nanobody-AuNP conjugate stability compared to physical adsorption; reproducible (intra-assay CV = 1.7%, inter-assay CV = 2.2%)	(24)
Streptavidin-bridged self-paired sandwich ELISA	double nanobody sandwich ELISA (Nb-ELISA), phage-mediated sandwich ELISA (p-ELISA) and SAB-ELISA	4.23×10^3 – 9.15×10^3 CFU/mL (six-fold improvement over traditional nanobody ELISA)	High specificity with negligible cross-reactivity to non-Salmonella pathogens	Multiplex detection, improved sensitivity and orientation, cost-effective nanobody production	(25)
Dual-mode immunochromatographic test strip (ITS) with colorimetric (AuNP signal) and photothermal (KMO@Au nanoflower + infrared laser readout) detection	Traditional ITS with monoclonal/polyclonal antibodies (less specific, higher background).	Colorimetric mode: 1×10^4 CFU/mL<br>Photothermal mode: 1×10^3 CFU/mL (10-fold better than colorimetric)	High specificity; negligible cross-reactivity with nine tested non-target pathogens (slight cross-reaction with S. Paratyphi due to LPS similarity)	Assay time: ~20 min<br>Visual naked-eye readout plus quantitative photothermal detection via portable infrared camera<br>Stable for at least 15 days (RSD ≤ 8.6%)<br>Portable, low-cost, on-site detection	(26)
Sandwich Enzyme-Linked Aptamer Sorbent Assay (ELASA)	NONE	95.74% (45 of 47 PCR-positive clinical isolates detected)	High, negligible cross-reactivity with E. coli, P. aeruginosa, S. aureus, M. catarrhalis, A. lwoffii, A. calcoaceticus, S. agalactiae	Rapid, low-cost assay<br>Does not require advanced instrumentation or costly facilities<br>Suitable for laboratory use, but not yet tested for field deployment	(18)
Dual-mode immunoassay: Colorimetric ELISA	Traditional mAb-ELISA: CELISA (0.09	CELISA LOD = 0.09 ng/mL SEA, linear range	No cross-reactivity with SEB, SEC, SED, or S. aureus protein A	Eliminates hazardous stop solutions (sulfuric acid)<br>Rapid, one-step detection with reduced reagents	(27)

(CELISA) + Fluorescent ELISA (FELISA)	ng/mL) and FELISA (0.40 ng/mL) showed ~16× higher sensitivity and simpler one-step workflow.<br>Commercial SEA kit: No significant difference ($p > 0.05$) in results for spiked milk/pork samples, confirming comparable diagnostic accuracy.	0.2–100 ng/mL<br>FELISA LOD = 0.40 ng/mL SEA, linear range 0.5–200 ng/mL<br>CELISA ~16× more sensitive than traditional antibody ELISA	High accuracy in spiked food matrices (milk, pork) with recoveries 93.63–104.96%, RSD <10%<br>Performance consistent with a commercial SEA kit	Dual-mode signals (colorimetric + fluorescent) provide mutual validation, improving reliability in complex samples<br>Environmentally friendly: quinine used as natural fluorescent product instead of synthetic dyes	
Nb-ELISA (conventional sandwich ELISA with nanobody)<br>P-ELISA (phage-mediated sandwich ELISA, phage-displayed nanobody as detection probe)<br>P-CLISA (phage-mediated chemiluminescent ELISA with nanobody capture + phage nanobody detection + HRP chemiluminescence readout)	Reference comparator: Plate-counting (culture method).<br>Internal comparators: Nb-ELISA and P-ELISA formats.	In spiked food matrices (juice, honey, milk, pork), the assay detected <10 CFU/mL after 6–8 h pre-enrichment, and results were consistent with plate-counting.	No cross-reactivity with nine non-target foodborne pathogens (S. aureus, C. albicans, C. coli, L. monocytogenes, V. parahaemolyticus, C. perfringens, E. coli O157:H7, etc)	Chemiluminescent readout improves sensitivity<br>Total assay time ~3 h (plus enrichment if required)<br>Reproducible (RSD ≤ 8.2%)<br>Applicable to diverse food samples.	(28)

Sandwich chemiluminescent immunoassay (CLIA)	Commercial SEB ELISA kit (recoveries 81.6–90.2%, no significant difference from CLIA).	Comparable to commercial ELISA; within regulatory standards (FDA threshold ~100 ng/mL).Working range: 3.12–50.0 ng/mL; SC_{50} (concentration giving 50% signal): 8.59 ± 0.37 ng/mL<br>Sensitivity comparison: The assay was reported to be comparable to a commercial SEB ELISA kit, but less sensitive than some advanced biosensors (LOD 0.001–0.01 ng/mL).	The Nb37–ALP CLIA showed high specificity:<br>No cross-reactivity with other staphylococcal enterotoxins (SEA),<br>No interference from bovine serum albumin (BSA), ovalbumin (OVA), or mouse IgG. Signals for these non-targets were equivalent to the blank control, confirming negligible cross-reactivity.	One-step immunoassay, avoids secondary antibody labeling, reduced assay time, thermally stable, cost-effective for scale-up.	(29)
Sandwich ELISA (three formats compared: mAbs-ELISA, Nbs-ELISA, OtTNb ELISA).	Directly compared with monoclonal antibody-based ELISA (mAbs-ELISA) and a commercial SEA ELISA kit	OtTNb ELISA was 3.4× more sensitive than mAbs-ELISA and 1.8× more sensitive than Nbs-ELISA. Compared to a commercial SEA kit, OtTNb ELISA had slightly lower absolute sensitivity (LOD ~3.8-fold higher) but a much broader linear range (8-fold wider).	No cross-reactivity with SEB, SEC, SED. Some cross-reactivity with SEE (expected, due to 70–90% sequence homology with SEA).<br>Resistant to Staphylococcal protein A (SpA) interference, unlike mAbs-ELISA, which produced false positives.	Simple ELISA format, compatible with standard lab equipment.<br>Stable nanobody (Nb26) under heat stress. Wide linear range, robust reproducibility (CVs 3.2–6.8%).	(30)

Nanobody-based sandwich ELISA coupled with immunomagnetic separation (IMS-ELISA)	pAb/Nb ELISA (~100× more sensitive); validated against culture plate-counting (consistent results)	Improved by 2 orders of magnitude compared to previous pAb/Nb ELISA. Detection range: 1.4×10^4 – 5.9×10^5 CFU/mL.	No cross-reactivity observed with other tested pathogens (E. coli, S. aureus, L. monocytogenes, Shigella, Vibrio spp., etc)	Rapid (detection within 4 h after enrichment). Good reproducibility (recoveries with CVs < 8%). Avoids matrix interference due to IMS.	(31)
ELISA (two types developed: biotinylated diVHH ELISA and diVHH-based sandwich ELISA)	traditional culture-based plate counting; results showed strong correlation (R^2 = 0.9056). Culture remains the gold standard but slower (~5 days vs. hours for ELISA).	High sensitivity in the sandwich ELISA format; linear correlation with culture-based plate counting (R^2 = 0.9056)	Recognized multiple Salmonella serotypes (Typhimurium, Enteritidis, Agalactiae, Dublin, Paratyphi B, Indiana, Anatum). No cross-reactivity with non-Salmonella tested species.	Faster than culture (detection within 1–6 h depending on enrichment), stable under harsh conditions (heat, pH), applicable to food matrices (lettuce, chicken, pork).	(32)
Nanobody-armed photothermal lateral flow immunoassay (NLFIA)	Compared with conventional AuNPs-LFIA (LOD 14.8 ng/mL → ~25× less sensitive). Benchmarked against ELISA, showing comparable or superior sensitivity.	ND	No cross-reactivity with SEA, SEC, SED, or Staphylococcal protein A (SpA). Demonstrated robustness against nonspecific interference.	Photothermal mode required a laser source + infrared camera, but enhanced sensitivity. Nanobody–CPNs showed stability at high ionic strength, pH extremes, and prolonged storage. Visual readout (colorimetric) still possible for simple applications.	(33)
One-step, label-free colorimetric biosensor using	NONE	Quantitative detection curve showed good	No cross-reactivity with 9 other pathogens tested (S. aureus, S. pyogenes, S. enteritidis,	Time-to-result: 100 min. One-step assay, visible readout, smartphone-capturable	(34)

thiolated phage-displayed nanobody (Phage-Nb-SH) + AuNP aggregation		linearity between 10^3–10^7 CFU/mL; assay stable with intra-day CV = 4.9% and inter-day CV = 9.45%	S. dysenteriae, E. coli, V. fluvialis, V. vulnificus, V. cholerae, etc.)	color change.<br>Low-cost platform using AuNPs and M13 phage	
Dual-mode nanobody immunoassay (colorimetric + fluorescence)	Compared with conventional antibody-based ELISA → RANbody dual-mode assay showed superior sensitivity and broader linear range	Linear Range: 0.31–2500 ng/mL.<br>Sensitivity: Higher than conventional antibody ELISA; practical detection in spiked food.	No cross-reactivity with SEA, SEC, SED, or S. aureus	Dual readout, simplified assay design, robust reproducibility, and compatibility with food matrices.	(35)
Sandwich ELISA	Culture (gold standard, slower); PCR (sensitive, costly, resource-intensive); conventional antibody ELISA (less stable, animal-dependent)	Detectable down to 10 CFU/mL in spiked milk after 8 h enrichment	No cross-reactivity with E. coli, Listeria monocytogenes, or Salmonella enteritidis	Faster and simpler than PCR or culture; enrichment required for very low detection limits; nanobody-based design reduces reliance on animals compared to polyclonal antibody production	(36)
Fe_3O_4@SiO_2@TQD dual-probe LFIA (lateral flow immunoassay) with nanobody–mAb probe combinations and test lines coated with monoclonal antibodies.	AuNP-based LFIA (traditional lateral flow, less sensitive). Dual-mAb ELISA (standard laboratory immunoassay, slower and less sensitive). Culture plate counting	Able to detect as low as 5 CFU in spiked milk and pork samples after enrichment.<br>Required enrichment time: SE (1.5 h), LM (2 h), CJ (6 h)	No cross-reactivity with Shigella, enterohemorrhagic E. coli (EHEC), Vibrio parahaemolyticus, Vibrio vulnificus, or Vibrio cholerae	Time-to-result: <15 min (much faster than ELISA 2–3 h and culture 1–3 days).<br>High throughput, portable, but fluorescence mode requires a fluorescence reader, limiting fully field-deployable use	(37)

	(gold standard, but time-consuming)				
Luminex-based multiplex bead assay (sandwich immunoassay)	Conventional monoclonal antibody (MAb 3b2a) and llama polyclonal IgG subclasses (IgG1, IgG2) Nanobody A3 alone or paired with MAb gave LOD 64 pg/mL, better than polyclonals; reverse pairing with MAb was weaker (40 ng/mL); nanobody more stable than conventional antibodies	sdAb A3 as capture + biotinylated MAb 3b2a detector: LOD ~64 pg/mL SEB.<br>Reverse (MAb capture + A3 detector): LOD ~40 ng/mL.<br>sdAb A3 alone as capture/detector: detection down to 64 pg/mL.	High specificity for SEB, no cross-reactivity with SEA, SED, Shiga toxin, ricin, or cholera toxin	Heat-stable sdAb allows potential for rugged field-deployable biosensors; retains binding after repeated heating/cooling cycles	(38)
Electrochemical immunosensor using screen-printed gold electrodes (AuSPE). Detection via voltammetric techniques (CV, DPV, SWV)	Genosensors: LoD 0.68 amol – 0.06 μg/mL, but stability less documented. Aptamer-based sensors: LoD 0.017 ng/mL – 33 CFU/mL. Antibody-based immunosensors: LoD	Strong linear relationship between current response and antigen concentration (R^2 ~0.96). LOD higher than advanced biosensors reported (0.001–0.2 ng/mL), but sufficient for clinical thresholds<br>CV: LoD = 3.1 ng/mL, R^2 =	No significant cross-reactivity with Listeria monocytogenes, Staphylococcus aureus, E. coli, or Salmonella enteritidis. Demonstrated selective response only to H. pylori antigen	Minimally invasive (saliva testing) Portable, cost-effective sensor platform Stable up to 24 days at RT Time-to-result short (standard voltammetry ~minutes)	(39)

	0.001 – 0.1 ng/mL; stability 70–94% at 4°C.<br>This Nb-sensor: Higher LoD (3–7 ng/mL), but better room-temperature stability and ease of construction.	0.960<br>DPV: LoD = 5.9 ng/mL, R^2 = 0.965<br>SWV: LoD = 7.2 ng/mL, R^2 = 0.963			
Colorimetric nanozyme assay (β-CD@AuNP peroxidase-like activity)	NONE	High sensitivity demonstrated; response was linear across tested concentration ranges with strong correlation coefficients ($R^2 > 0.95$)	Tested against non-target bacteria (E. coli, S. aureus, L. monocytogenes) — negligible signal change, confirming good selectivity. Selectivity ensured by β-CD functionalization which stabilized AuNPs and promoted catalytic response to Salmonella antigens	Rapid detection (minutes), simple colorimetric readout within minutes<br>Simple readout, visible colorimetric response (no advanced instrumentation required).<br>Cost-effective, avoids the use of antibodies and enzymes, instead utilizing nanozymes.<br>Matrix compatibility, worked in lettuce/meat spiking experiments	(40)
ELISA 1: Streptavidin-bridged double nanobody ELISA.<br>ELISA 2: Nanobody–HRP direct fusion ELISA (avoids secondary antibody).<br>TRFICA: Time-resolved fluorescence immunochromatographic	Compared against mouse bioassay (gold standard for BoNT). Also contextualized against traditional antibody-based ELISAs.<br>TRFICA demonstrated comparable LOD to	TRFICA: Sensitivity 98.0%, Specificity 96.7% (in clinical samples).<br>ELISAs: High sensitivity but slower (longer incubation steps)	No cross-reactivity between BoNT/A and BoNT/B assays	ELISA: ~100 min detection time, requires laboratory equipment, stable in complex matrices.<br>TRFICA: ~15 min, portable, suitable for field screening, but more prone to interference from hemolyzed or clotted serum.	(17)

assay using lanthanide nanospheres.	mouse bioassay but with shorter turnaround				
Magnetofluorescent nanobody-based sandwich assay	Compared to conventional ELISA (LOD ~10^4 CFU/mL)	Very high sensitivity; reliable detection of E. coli F17 in culture and spiked fecal samples, recovery 89–102%	High; no cross-reactivity with E. coli BL21-DE3, Salmonella, Listeria, Staphylococcus	Time to result = ~90 min. Stability: assay maintained ≥90% activity after 30 days at 4 °C. Usable directly in untreated fecal samples.	(41)
Phage-mediated triple antibody sandwich immunoassay (TAS-ELISA)	mAb-ELISA (standard antibody-based ELISA). Plate counting (used for validation in spiked milk)	Compared to conventional methods: TAS-ELISA was 41.8-fold more sensitive than mAb-ELISA. TAS-ELISA was about 9-fold more sensitive than DAS-ELISA (single nanobody). Sensitivity confirmed by recovery values in spiked milk (83.3–90.7% intra-assay; 76.3–105.2% inter-assay; CV values < 9.46% demonstrated good reproducibility)	TAS-ELISA: Strong specific response only to E. coli O157:H7. No cross-reactivity with other E. coli serotypes or unrelated bacteria. mAb-ELISA showed slight cross-reactivity with E. coli O103:H2.	Time: Comparable to ELISA (~several hours). Cost: Potentially higher than mAb-ELISA due to nanobody phage-display system, but lower than molecular methods (PCR). Ease of use: Similar to conventional ELISA, adaptable to food testing laboratories. Stability: TAS-ELISA stable for at least 20 days at 4°C without performance loss	(42)

Nanobody-based sandwich ELISA (Nbs-ELISA)	NONE	The assay showed a linear quantitative detection range of 4–250 ng/mL. Recovery rates in spiked dairy products: 84.5–108% with CVs between 2.5–11.9%.	Minimal cross-reactivity with SEA, SEB, SpA, and S. aureus strains (ATCC 25923, 29213)	Time to result: Similar to standard ELISA (~3–4 hours including incubation/wash cycles). Ease of use: Standard ELISA workflow; no need for complex instrumentation. Cost: Likely lower than monoclonal antibody-based ELISA due to simple nanobody production in E. coli. Matrix tested: Fresh milk, yogurt, cheese – method performed with good accuracy in real dairy matrices.	(43)
Sandwich ELISA and direct ELISA	Conventional antibody-based ELISA (mAb/pAb pairs) was used as benchmark	190–390 pg/mL	Nanobody-based ELISAs were highly sensitive, with detection limits in the picogram range (190–390 pg/mL) for SEB in milk. Maintained sensitivity even after heat treatment of nanobodies (up to 95°C), unlike conventional antibodies which showed reduced binding	Food safety detection in dairy; lab-based; stable under heat; not validated in naturally contaminated samples	(44)
double-VHH sandwich ELISA for Stx2 detection	Commercial capture ELISA (R-Biopharm). Commercial rapid immunoassay (Shiga Toxin Quik Chek, Alere-Abbott). PCR for stx2 genes	Clinical sensitivity was comparable to PCR and the Shiga Toxin Quik Chek (Alere/Abbott) rapid test	The selected VHH pair (1vb1–2vb10) showed high specificity for Stx2a. No cross-reactivity was observed with Stx1. Specificity was comparable to PCR and Shiga Toxin Quik Chek — very few false positives were reported.	Tested on clinical stool samples from patients with diarrhea, bloody diarrhea, and HUS. Showed high concordance with PCR and commercial rapid tests . Potential extension to rapid 10-min immunochromatographic format for point-of-care settings	(45)
sandwich ELISA-based quantification assays for TcdA and TcdB.	Benchmarked against conventional ELISA readouts and toxin	TcdA (A2B10/A1A6 pair): LOD 0.6 ng/mL (buffer/supernatant), 12	Nanobody ELISAs specifically detected TcdA or TcdB without cross-reactivity to unrelated proteins.	Rapid ELISA format with HRP–streptavidin signal readout. Good sensitivity in purified/culture samples, reduced sensitivity in biological samples (matrix effect).	(46)

	levels measured in different strains and matrices	ng/mL (feces/cecal content).<br>TcdA (A1D8/A1C3 pair): LOD 0.019 ng/mL (buffer), 0.075 ng/mL (feces), 0.6 ng/mL (cecal).<br>TcdB (B2C11/B0E2 pair): LOD 2.1 ng/mL (buffer/supernatant).<br>TcdB (B0D10/B0E2 pair): LOD 0.033 ng/mL (buffer), 0.65 ng/mL (supernatant), but failed in feces.	Epitope mapping confirmed binding to distinct domains (CROPs, GTD, DD), supporting domain-specific specificity.	Provides a toolkit for toxin quantification in infection models.	
Double nanobody-based sandwich ELISA (self-paired) for detection of S. Enteritidis in food (milk) and in vivo (chicken organs)	Real-time PCR and plate counting (validation in chicken tissues)	LOD (Limit of Detection): 5×10^4 CFU/mL Any sample with an OD450 nm > 0.188 was considered positive, corresponding to $\geq 5 \times 10^4$ CFU/mL of S. Enteritidis.	No cross-reactivity reported; negative control wells remained blank	Requires 8 h pre-enrichment; ELISA run ~3 h; cost-effective and user-friendly compared to PCR; applicable to food testing and in vivo studies	(47)

Table S3. Summary of the main detection methods evaluated across 32 studies selected, according to their sensitivity, specificity, and overall practicality in detection applications
